# Multiple attribute group decision-making based on interval-valued q-rung orthopair uncertain linguistic power Muirhead mean operators and linguistic scale functions

**DOI:** 10.1371/journal.pone.0258772

**Published:** 2021-10-21

**Authors:** Yuan Xu, Shifeng Liu, Jun Wang

**Affiliations:** 1 School of Economics and Management, Beijing Jiaotong University, Beijing, China; 2 School of Economics and Management, Beijing University of Chemical Technology, Beijing, China; University of Defence in Belgrade, SERBIA

## Abstract

Fuzzy set theory and its extended form have been widely used in multiple-attribute group decision-making (MAGDM) problems, among which the interval-valued q-rung orthopair fuzzy sets (IVq-ROFSs) got a lot of attention for its ability of capturing information denoted by interval values. Based on the previous studies, to find a better solution for fusing qualitative quantization information with fuzzy numbers, we propose a novel definition of interval-valued q-rung orthopair uncertain linguistic sets (IVq-ROULSs) based on the linguistic scale functions, as well as its corresponding properties, such as operational rules and the comparison method. Furthermore, we utilize the power Muirhead mean operators to construct the information fusion method, and provide a variety of aggregation operators based on the proposed information description environment. A model framework is constructed for solving the MAGDM problem utilizing the proposed method. Finally, we illustrate the performance of the new method and investigate its advantages and superiorities through comparative analysis.

## 1 Introduction

MAGDM is essentially a process of making choices from a set of alternatives based on multiple decision makers’ (DMs’) evaluations under several attributes, whose methods and theories have been rapidly development in the past few decades [1–10]. In recent years, As the complexity of real-world problems increases, DMs’ expressions of evaluation decisions are becoming more and more diversified and complicated. Therefore, how to describe the evaluation information of DMs more accurately has become a topic deserving research. In response to this problem, a widely accepted approach is to apply fuzzy theory [11] to MAGDM problems, by fuzzifying and then defuzzifying the uncertain semantic contexts, the complex semantic information can be included as comprehensively as possible. Among the classical fuzzy theory family—intuitionistic fuzzy sets (IFSs) [12], Pythagorean fuzzy sets (PFSs) [13], and q-rung orthopair fuzzy sets (q-ROFSs) [14]—the q-ROFSs is particularly outstanding because it requires that the *q*th sum of the membership degree (MD) and the non-membership degree (NMD) is no larger than one.

The relatively broader restriction provides q-ROFSs a solid position in the application of evaluation information representation [15,16]. However, the shortcoming of q-ROFSs is also obvious, the fact that the MD and NMD can only be expressed by a crisp number value suppresses its applicability in the situations of insufficient information and obvious hesitation of DMs. Therefore, one of the research questions is clearly defined as: How to improve the applicability of q-ROF in a highly complex and uncertain decision-making environment? When dealing with MAGDM problems under IVq-ROULSs, the construction of information fusion method by AOs is also an important part. Thus, another research question is: How to effectively aggregate the evaluation information expressed by interval-valued q-rung orthopair uncertain linguistic variables (IVq-ROULVs)?.

To further provide more decision-making freedom for DMs, many scholars have proposed new information expression methods on the basis of q-ROFSs. For example, Xu et al. [17] incorporated the dual hesitant fuzzy set into q-ROFS and proposed the q-rung dual hesistant fuzzy sets (q-RDHFSs); Joshi et al. [18] applied interval values to the MDs and NMDs of q-ROFSs and proposed the IVq-ROFSs; Xu et al. [19] gave a novel definition of interval-valued q-RDHFSs (IVq-RDHFSs); Moreover, some scholars applied the q-ROFSs with linguistic terms sets and uncertain linguistic term sets to the research of qualitative quantization methods, such as the linguistic q-ROFSs [20–22], the q-rung picture linguistic sets [23], q-rung orthopair uncertain linguistic sets (q-ROULSs) [24–27], etc. Utilizing q-ROULSs, DMs can quantify qualitative evaluation information through uncertain linguistic values (ULVs). However, in most real MAGDM problems, DMs preferer to employ interval values to express themselves. Similar researches have already conducted [28,29]. On this basis, this paper extends the q-ROULSs to interval-valued q-ROULSs (IVq-ROULSs), which not only allows experts to employ interval-valued q-rung orthopair fuzzy numbers in the representation of MDs and NMDs, but also can utilize ULVs to express qualitative valuation. We elaborate on the superiority of the proposed fuzzy set as follows: First, compared with q-ROULSs, the IVq-ROULSs contain more information and so that they can comprehensively express DMs’ evaluation values. In addition, the IVq-ROULS is an extension of the q-ROULS, indicating that the former fuzzy set is more powerful and flexible than the later one. Second, the IVq-ROULS is essentially a hybrid, so it has the advantages of both IVq-ROFS and ULVs, and performs better than IVq-ROFS in coping with qualitative evaluations. Finally, the IVq-ROULSs are also more powerful than IVIULSs and IVPULSs, because IVq-ROULSs can provide DMs more relaxed evaluation restrictions and release decision-making freedom. In the following, we utilize a short example to better illustrate the advantages of the proposed method:

Suppose that a DM gives an evaluation represented by <(s_2_, s_4_), ([0.5, 0.7], [0.6, 0.8])>. It cannot be processed by q-ROULSs, because its MD and NMD are expressed with interval-values; It cannot be processed by IVIULSs, because the sum of the upper values of MD and NMD is 1.5 and larger than 1; It cannot be processed by IVPULSs, because the square sum of the upper values of MD and NMD is 1.13 and larger than 1. However, it can be processed with the proposed IVq-ROULSs. More details can be found in subsection 4.2.

As for the information fusion method, the power average (PA) operator originated by Prof. Yager [30] is an efficient information aggregation technology. Due to its characteristic that it can reduces the damage of inappropriate extreme evaluation information to the results, the PA has received much attention globally [31–34]. This paper extends the PA operator to IVq-ROULSs and introduces several information fusion methods, i.e., the IVq-ROUL PA operator and its weighted form (IVq-ROULPWA). In reality, the cross-relationship among attributes occupies an important position in the evaluation of MAGDM. The power Muirhead mean (PMM) [35], which is an integration of the PA and Muirhead mean [36] operators, is a strong AO introduced by Li and her colleagues. This operator has the function of capturing the relationship among attributes and reducing the influence of extreme values simultaneously [37–41]. In this paper, we further utilize the PMM operator and its weighted form to aggregate variables represented by IVq-ROUL architectures. Therefore, the IVq-ROUL PMM operator and its weighted form are proposed as information fusion operators as well. Finally, a complete model is introduced to show the process of coping with MAGDM problems with high degree of complexity, and the advantages of the proposed method are illustrated by numerical experiments.

Based on the above analysis, the novelty can be attributed to the following: First, to improve the applicability of q-ROFSs in a highly complex and uncertain decision-making environment, this paper proposes a novel MAGDM information expression method based on IVq-ROULSs. Then, two information fusion operators IVq-ROUL PMM and its weighted form were provided to aggregate the evaluation information of MAGDM. Finally, a comprehensive novel model to MAGDM with interval-valued q-rung orthopair uncertain linguistic information are derived. To sum up, the motivations and contributions of this paper can be briefly summarized as: (1) To comprehensively express DMs’ evaluation values, it is necessary to establish an information expression model from both quantitative and qualitative aspects. With the proposed IVq-ROULSs, DMs can express their qualitative evaluations based on a pre-defined LTS and also can provide the MDs and NMDs constructed with interval-valued q-rung orthopair fuzzy numbers, which are quantitative information. (2) To effectively aggregate DMs’ evaluation information expressed by IVq-ROULVs, this paper proposes the operational rules and then give the definition of the IVq-ROUL PMM operator and its weighted form. Not only can the new proposed operator consider multiple interrelationships among attributes, but it can also reduce the negative impacts of extreme evaluation values. (3) To demonstrate the application effect of the proposed method, a comprehensive application model is given. Compared with the existing MAGDM methods, our method has much broader constraints, stronger robustness, wider range of use, and superior flexibility.

The rest part of this paper is organized as follows. Section 2 reviews the related works. Section 3 reviews the definitions of IVq-ROFSs and some AOs. Section 4 introduces the detailed motivations, then gives the definition of IVq-ROULSs and the corresponding operational rules, comparisons method and distance measure method. Section 5 presents a series of IVq-ROUL operators, their properties and some special cases are given as well. Section 6 presents a complete model of MAGDM method under IVq-ROULSs. In Section 7, the application process of the proposed method is introduced in detail and its advantages are illustrated through comparative analysis. Section 8 concludes the whole paper.

## 2 Related works

To overcome the shortcomings of IFSs and PFSs, recently, Prof. Yager [14] proposed the q-ROFSs, satisfying the constraint that *u*+*v*∈[0,1], where *u* and *v* denote the membership-degree (MD) and non-membership degree (NMD), respectively. Compared with IFSs and PFSs, the q-ROFSs have laxer constraint and more powerful because its wider range of applications. Since put forward, more and more aggregation operators (AOs), such as, weighted averaging operator [42,43], Bonferroni mean operator [44,45], Hamy mean operator [46,47], Heronian mean operators [48], Maclaurin symmetric mean operator [49,50], Muirhead mean [51], Choquet integral operator [52], power weighted aggregation operator [53], etc., are employed to fusion the evaluation information described by q-ROF numbers (q-ROFNs). In addition, some other scholars focused on operational laws of q-ROFNs. For instance, Jana et al. [54] proposed new q-ROF operation under Dombi t-norm and t-conorm. Liu and Wang [55] investigated novel operations of q-ROFNs under Archimedean t-norm and t-conorm. Xing et al. [56] introduced the interactive operations of q-ROFNs, which consider the interaction between MDs and NMDs. Peng et al. [57] presented exponential operations of q-ROFNs and investigated their applications in decision-making. The above researches proves that q-ROFSs theory has favorable application effect in MAGDM problems.

It should be noted that the above researches are mainly based on the q-ROFSs. In addition, some scholars have focused on studying the extension of q-ROFSs. For instance, Xu et al. [17] extended q-ROFSs to q-RDHFSs, which can utilize multiple evaluation values to represent the MDs and NMDs, and it’s an effective information expression method that can solve situations where DMs are hesitant among several estimates. Influenced by the same principle, Joshi et al. [18] proposed the IVq-ROFSs, which have an excellent performance in dealing with DMs’ ambiguity and fuzziness in the process of decision-making. Although Xu et al.’s [17] and Joshi et al.’s [18] methods both have extended q-ROFSs’ ability to process uncertain information, they cannot solve the problem whose information are expressed by the other party’s method. To fill this gap, Xu et al. [19] gave the definition of IVq-RDHFSs, whose MDs and NMDs are expressed by several numbers construct with interval-values, which can be regarded as a generalized form of Xu et al.’s [17] and Joshi et al.’s [18] methods, it can be degenerated into any of the two under the action of different parameters. In addition, Garg [58] proposed the connection number-based q-rung orthopair fuzzy set by incorporating the q-ROFSs and the connection number. Garg et al. [59] introduced the complex interval-valued q-rung orthopair fuzzy set to better express the time-periodic problems and two-dimensional information in a single set. More related works of extension of q-ROFSs can refer to [60,61].

Recently, Zhang [62] applied linguistic terms to IFSs and proposed the linguistic IFSs, which is characterized by expressing both qualitative and quantitative information through one linguistic MD and one linguistic NMD. Since then, the fuzzy quantitative expression of semantic information has been widely concerned. Subsequently, Garg [63] provides the concept of linguistic PFSs and applied it to MAGDM problems. P. Liu and W. Liu [20] combined linguistic terms with q-ROFSs and proposed the linguistic q-rung orthopair fuzzy number (Lq-ROFN), Compared with Zhang’s [62] and Garg’s [63] methods, Lq-ROFN can be regard as a generalization that preserves DMs’ evaluation freedom to the greatest extent possible. Although the above studies have provided a solution to the fuzzy representation of qualitative evaluation information to a certain extent, they are still insufficient for conditions containing uncertain semantic information. Therefore, to strengthen the express ability of uncertain linguistic information, Wang et sl. [24] integrated uncertain linguistic variables into q-ROFSs and proposed the q-rung orthopair uncertain linguistic sets (q-ROULSs). Xing et al. [25], Liu et al. [26], and Bai et al. [27] further conducted extended studies on this method. Furthermore, some scholars incorporate interval-values into ULVs, Liu [28] extended the intuitionistic uncertain linguistic sets (IULSs) to interval-valued intuitionistic uncertain linguistic sets (IVIULSs) to allow DMs provide their evaluation information with interval values. Gao and Wei [29] further proposed the interval-valued Pythagorean uncertain linguistic sets (IVPULSs). Since proposed, researches based on IVIULSs and IVPULSs have become popular research fields [64,65].

Although the methods based on IVIULSs and IVPULSs have been illustrated their effectiveness in MAGDM problems many times, they are constructed based on IFSs and PFSs. And thus, they have the inherent shortcomings just as IFSs and PFSs, namely, overly restrictive conditions on MDs and NMDs. In addition, as analyzed in Section 1, q-ROFSs is a powerful tool to fill this gap. Therefore, in this paper, we aim to extend the q-ROULSs to interval-valued q-ROULSs (IVq-ROULSs), which not only allows experts to employ interval-valued q-rung orthopair fuzzy numbers in the representation of MDs and NMDs, but also can utilize ULVs to express qualitative valuation. Based on this, a new MAGDM method is proposed, which is theoretically more flexible, freer and more precise than all the above methods.

## 3 Basic concepts

The aim of this section is to recall some basic notions, such as IVq-ROFSs, the PA, MM and PMM operators.

### 3.1 The interval-valued q-rung orthopair fuzzy set

**Definition 1** [18]: Let *X* be an ordinary fixed set, an interval-valued q-rung orthopair fuzzy set (IVq-ROFS) defined on *X* is expressed as

$$A=\{\left\langle x,\mu_{A}(x),v_{A}(x)\rangle |x\in X\right\},$$ (1)

where *μ*_*A*_:*X*→[0,1] and *v*_*A*_:*X*→[0,1] are two interval values, denoting the MD and NMD, respectively, with the constraint that (sup(*μ*_*A*_(*x*)))^*q*^+(sup(*v*_*A*_(*x*)))^*q*^≤1. Then the hesitancy degree is expressed as

$$\pi_{A}(x)={\left(1-{\left(\mu_{A}(x)\right)}^{q}-{\left(v_{A}(x)\right)}^{q}\right)}^{1/q},$$ (2)

where *π*_*A*_(*x*) is an interval number in [0, 1]. For convenience, the ordered pair *A* = (*μ*_*A*_(*x*),*v*_*A*_(*x*)) is called an interval-valued q-rung orthopair fuzzy number (IVq-ROFN), which can be denoted as *α* = ([*a*,*b*],[*c*,*d*]), satisfying the conditions [*a*,*b*],[*c*,*d*]⊂[0,1] and *b*^*q*^+*d*^*q*^≤1(*q*≥1).

Furthermore, Gao et al. [66] introduced a method to rank any two IVq-ROFNs.

**Definition 2** [66]: Let *α* = ([*a*,*b*],[*c*,*d*]) be an IVq-ROFN, then the score function of is defined as

$$S(\alpha )=\frac{1+a^{q}-c^{q}+1+b^{q}-d^{q}}{4},$$ (3)

and the accuracy function *H*(*α*) of *α* is

$$H(\alpha )=\frac{a^{q}+b^{q}+c^{q}+d^{q}}{2}.$$ (4)

Let *α*_1_ = ([*a*_1_,*b*_1_],[*c*_1_,*d*_1_]) and *α*_2_ = ([*a*_2_,*b*_2_],[*c*_2_,*d*_2_]) be any two IVq-ROFNs, then

1. If *S*(*α*_1_)>*S*(*α*_2_), then *α*_1_ = *α*_2_;
2. If *S*(*α*_1_) = *S*(*α*_2_), then
  if *H*(*α*_1_)>*H*(*α*_2_), then *α*_1_>*α*_2_;
  if *H*(*α*_1_) = *H*(*α*_2_), then *α*_1_ = *α*_2_

The operations of IVq-ROFNs are presented in [18,66].

**Definition 3** [18,66]: Let *α*_1_ = ([*a*_1_,*b*_1_],[*c*_1_,*d*_1_]), *α*_2_ = ([*a*_2_,*b*_2_],[*c*_2_,*d*_2_]) and *α* = ([*a*,*b*],[*c*,*d*]) be three IVq-ROFNs and *λ* be a positive real number, then

1. $\alpha_{2}⊕\alpha_{2}=(\left[{\left(a_{1}^{q}+a_{2}^{q}-a_{1}^{q}a_{2}^{q}\right)}^{1/q},{\left(b_{1}^{q}+b_{2}^{q}-b_{1}^{q}b_{2}^{q}\right)}^{1/q}],[c_{1}c_{2},d_{1}d_{2}\right])$;
2. $\alpha_{2}⊗\alpha_{2}=(\left[a_{1}a_{2},b_{1}b_{2}],[{\left(c_{1}^{q}+c_{2}^{q}-c_{1}^{q}c_{2}^{q}\right)}^{1/q},{\left(d_{1}^{q}+d_{2}^{q}-d_{1}^{q}d_{2}^{q}\right)}^{1/q}\right])$;
3. $\alpha^{\lambda }=(\left[{\left(1-{\left(1-a^{q}\right)}^{\lambda }\right)}^{1/q},{\left(1-{\left(1-b^{q}\right)}^{\lambda }\right)}^{1/q}],[c^{\lambda },d^{\lambda }\right])$;
4. $\lambda \alpha =(\left[a^{\lambda },b^{\lambda }],[{\left(1-{\left(1-c^{q}\right)}^{\lambda }\right)}^{1/q},{\left(1-{\left(1-d^{q}\right)}^{\lambda }\right)}^{1/q}\right])$.

### 3.2 The power average, Muirhead mean and power Muirhead mean operators

In real MAGDM problem, the decision-making information provided by DMs maybe unduly high or low because of their different backgrounds and individual preferences. To reduce the negative impact of extreme evaluations on decision-making results, Prof. Yager [30] introduced PA operator. The definition of PA is presented as follows.

**Definition 4** [30]: Let *a*_*i*_(*i* = 1,2,…,*n*) be a collection of positive real numbers, then the power average (PA) operator is defined as

$$PA(a_{1},a_{2},\ldots ,a_{n})=\frac{\sum_{i=1}^{n}(1+T(a_{i}))a_{i}}{\sum_{i=1}^{n}(1+T(a_{i}))},$$ (5)

where $T(a_{i})=\sum_{j=1,j\neq i}^{n}Sup(a_{i},a_{j})$ and *Sup*(*a*_*i*_,*a*_*j*_) denotes the support degree for *a*_*i*_ from *a*_*j*_, satisfying the following conditions: 1) *Sup*(*a*_*i*_,*a*_*j*_)∈[0,1]; 2) *Sup*(*a*_*i*_,*a*_*j*_) = *Sup*(*a*_*j*_,*a*_*i*_); 3) *Sup*(*a*,*b*)≥*Sup*(*c*,*d*), if |*a*−*b*|≤|*c*−*d*|.

The MM operator introduced by Muirhead [36] receives great reputation for its ability of capturing the interrelationship among any numbers of input arguments.

**Definition 5** [36]: Let *a*_*j*_(*j* = 1,2,…,*n*) be series of positive real numbers, and *H* = (*h*_1_,*h*_2_,…,*h*_*n*_)∈*R*^*n*^ be a set of parameters. If

$$MM^{H}(a_{1},a_{2},\ldots ,a_{n})={\left(\frac{1}{n!}\sum_{\zeta \in T_{n}}\prod_{j=1}^{n}a_{\zeta (j)}^{h_{j}}\right)}^{\frac{1}{\sum_{j=1}^{n}h_{j}}},$$ (6)

then *MM*^*H*^ is called the MM operator, where *T*_*n*_ denotes all permutations of (1,2,…,*n*) and *ς*(*j*)(*j* = 1,2,…,*n*) is any permutation of (1,2,…,*n*).

Combined PA with MM operator, Li et al. [35] proposed the PMM operator.

**Definition 6** [35]: Let *a*_*j*_(*j* = 1,2,…,*n*) be a collection of crisp numbers and *H* = (*h*_1_,*h*_2_,…,*h*_*n*_)∈*R*^*n*^ be a vector of parameters. The PMM operator is defined as follows:

$$PMM^{H}(a_{1},a_{2},\ldots ,a_{n})={\left(\frac{1}{n!}\sum_{\zeta \in T_{n}}\prod_{j=1}^{n}{\left(\frac{n(1+T(a_{\zeta (j)}))a_{\zeta (j)}}{\sum_{j=1}^{n}\left(1+T(a_{j})\right)}\right)}^{h_{j}}\right)}^{\frac{1}{\sum_{j=1}^{n}h_{j}}},$$ (7)

where *T*_*n*_ denotes all permutations of (1,2,…,*n*) and *ς*(*j*)(*j* = 1,2,…,*n*) is any permutation of (1,2,…,*n*). $T(a_{j})=\sum_{i=1,i\neq j}^{n}Sup(a_{i},a_{j})$ and *Sup*(*α*_*i*_,*α*_*j*_) denotes the support degree for *α*_*i*_ from *α*_*j*_, satisfying the following properties presented in Definition 4.

## 4 The interval-valued q-rung orthopair uncertain linguistic sets

In this section, we introduce the concept of IVq-ROULSs. Some other related notions are also presented.

### 4.1 Motivations of proposing the IVq-ROULS

In practical decision-making problems, to comprehensively express DMs’ evaluation values, it is necessary to establish an information expression model from both quantitative and qualitative aspects. Basically, DMs are required to express their qualitative evaluations based on a pre-defined LTS and in addition, they should also provide the MDs and NMDs, which are quantitative information. In light of this, the IVIULSs and IVPULSs are two effective tools, which can effectively denote DMs’ assessments. However, the IVIULSs and IVPULSs still have shortcoming. We provide the following example to illustrate the drawback of IVIULSs and IVPULSs.

**Example 1.** Three professors are invited to evaluate the research ability of a student. Let *S* = {*s*_0_ = “very low”, *s*_1_ = “low”, *s*_2_ = “slightly low”, *s*_3_ = “medium”, *s*_4_ = “slightly good”, *s*_5_ = “good”, *s*_6_ = “very good”} be a given LTS. Each DM is required to use an ULV over *S* to denote his/her qualitative evaluation. In addition, every DM should provide two interval values, to depict the MD and NMD of the ULV provide by himself/herself. The evaluation information of the three professors is listed in Table 1. The evaluation values of the three professors can be denoted as *α*_1_ = 〈[*s*_4_,*s*_5_],([0.5,0.6],[0.2,0.4])〉, *α*_2_ = 〈[*s*_3_,*s*_5_],([0.4,0.7],[0.5,0.6])〉 and *α*_3_ = 〈[*s*_4_,*s*_6_],([0.6,0.9],[0.5,0.8])〉, respectively. Obviously, as 0.6 + 0.4 = 1, *α*_1_ is an interval-valued intuitionistic uncertain linguistic variable (IVIULV). The evaluation value *α*_2_ can be handled by IVPULSs as 0.7 + 0.6 = 1.3 > 1 and 0.7^2^ + 0.6^2^ = 0.85 < 1. However, the evaluation value *α*_3_ cannot be handled by either IVULSs or IVPULSs, as 0.9 + 0.8 = 1.7 > 1 and 0.9^2^ + 0.8^2^ = 1.45 > 1. Hence, the IVIULSs and IVPULSs are still insufficient to deal with some complex decision-making situations. In fact, IVIULSs and IVPULSs have their own theoretical limitations on the MDs and NMDs, the former set requires that the sum of that is no larger than one, the latter one requires that the square sum of that is no larger than one. But, these two constraints cannot be always strictly satisfied. Hence, it is necessary to provide a novel method with laxer restrictions. Given the laxer constraint of IVq-ROFSs that the sum of *q*th power of MD and *q*th power of NMD should be less than or equal to one, we propose the IVq-ROULSs by combining IVq-ROFSs with LTS. Obviously, the proposed IVq-ROULSs are more empowered and can depict more complicated decision information than IVIULSs and IVPULSs.

**Table 1:** The evaluation values provided by the three professors.

	The ULV	The MD	The NMD
The first professor	[*s*_4_, *s*_5_]	[0.5, 0.6]	[0.2, 0.4]
The second professor	[*s*_3_, *s*_5_]	[0.4, 0.7]	[0.5, 0.6]
The third professor	[*s*_4_, *s*_6_]	[0.6 0.9]	[0.5, 0.8]

### 4.2 Definition of IVq-ROULS

**Definition 7.** Let *X* be an ordinary set and $\bar{S}$ be a continuous LTS of *S* = {*s*_*i*_|*i* = 1,2,…,*t*} with odd cardinality, then an interval-valued q-rung orthopair uncertain linguistic set (IVq-ROUL) set *A* defined on *X* is expressed as

$$A=\{\left\langle x,[s_{\theta (x)},s_{\tau (x)}],(\mu_{A}(x),v_{A}(x))\rangle |x\in X\right\},$$ (8)

where $[s_{\theta (x)},s_{\tau (x)}]\in \bar{S}$, *μ*_*A*_:*X*→[0,1] and *v*_*A*_:*X*→[0,1] are two interval values, denoting the MD and NMD of the element *x*∈*X* to the set *A*, respectively, satisfying the condition ${\left(\sup(\mu_{A}(x))\right)}^{q}+{\left(\sup(v_{A}(x))\right)}^{q}\leq 1,(q\geq 1)$. For convenient description, we call $\left\langle \left[s_{\theta (x)},s_{\tau (x)}],(\mu_{A}(x),v_{A}(x)\right)\right\rangle$ an IVq-ROULV, which can be denoted as *α* = 〈[*s*_*θ*_,*s*_*τ*_],([*a*,*b*],[*c*,*d*])〉 for simplicity.

From Definition 7, we can find out that when *q* = 1, the IVq-ROULSs reduce to the IVIULSs. When *q* = 2, the IVq-ROULSs reduce to IVPULSs. In Example 1, the evaluation value *α*_3_ is an IVq-ROULV, as 0.9^5^ + 0.8^5^ = 0.9182 < 1. In addition, the evaluation values *α*_1_ and *α*_2_ can be also regarded as IVq-ROULVs, which also illustrates the powerfulness and flexibility of IVq-ROULSs.

### 4.3 Operational rules of IVq-ROULVS

In the following, we propose some basic operations of IVq-ROULVs. Before doing so, we first review the concept of linguistic scale function (LSF).

**Definition 8** [67]: Let *S* = {*s*_*i*_|*i* = 0,1,…,2*t*} be an LTS, *s*_*i*_∈*S* be a linguistic variable. For any real number *γ*_*i*_(*i* = 0,1,2,…,2*t*), a LSF *f* is a mapping from *s*_*i*_ to *γ*_*i*_(*i* = 0,1,2,…,2*t*) such that:

$$f:s_{i}\to \gamma_{i}(i=0,1,2,\ldots ,2t),$$

where 0≤*γ*_0_<*γ*_1_<…<*γ*_2*t*_.

The three most widely used LSFs are presented as follows.


$$\mathrm{LSF}1:f_{1}(s_{i})=\gamma_{i}=\frac{i}{2t}(i=0,1,2,\ldots ,2t)$$ (9)

$$\mathrm{LSF}2:\;f_{2}(s_{i})=\gamma_{i}=\{\begin{array}{l} \frac{\rho^{t}-\rho^{t-i}}{2\rho^{t}-2}\;(i=0,1,2,\ldots ,t)\\ \frac{\rho^{t}+\rho^{i-t}-2}{2\rho^{t}-2}(i=t+1,t+2,\ldots ,2t) \end{array},$$ (10)

$$\mathrm{LSF}3:\;f_{3}(s_{i})=\gamma_{i}==\{\begin{array}{l} \frac{t^{\varepsilon }-{\left(t-i\right)}^{\varepsilon }}{2t^{\varepsilon }}\;(i=0,1,2,\ldots ,t)\\ \frac{t^{\beta }+{\left(i-t\right)}^{\beta }}{2t^{\beta }}\;(i=t+1,t+2,\ldots ,2t) \end{array},(\varepsilon ,\beta \in [0,1])\;$$ (11)

For Eq (10), The value of *ρ* is given by the subjective judgment of experts, the *γ*_*i*_ and the absolute deviation between two adjacent linguistics increases. Assume that the input argument *A* ahs greater weight than input argument *B* (suppose that *m* represents the weight ratio, and the scale level is expressed by *k*), then we can know that *ρ*^*k*^ = *m* and $\rho =\sqrt[{k}]{m}$. Most researchers consider that 9 is an appropriate value to be regard as the upper limit of the weight ratio. Therefore, when the scale level is 7, we can obtain $\rho =\sqrt[{7}]{9}\approx 1.37$. More detailed information can be found in [67].

Especially, Eq (9) is a special case of Eq (11) when *ε* = *β* = 1. In addition, the function *f* can also be extended as a continuous function, e.g., $f*:\tilde{f}\to \Omega^{+}(\Omega^{+}=\{d|d\geq 0\},d\in R)$ with f*(*s*_*i*_) = *γ*_*i*_. Utilize f*^−1^ to represent the inverse function of f*. Then, we can get

$$\mathrm{LSF}1:\;f_{1}{*}^{-1}(\gamma_{i})=s_{2t*i}(i=0,1,2,\ldots ,2t),$$ (12)

$$\mathrm{LSF}2:\;f_{2}{*}^{-1}(\gamma_{i})=\{\begin{array}{l} s_{t-\log_{\rho }(\rho^{t}-(2\rho^{t}-2)\gamma_{i})},\;(\gamma_{i}\in [0,0.5])\\ s_{t+\log_{\rho }((2\rho^{t}-2)\gamma_{i}-\rho^{t}+2)},\;(\gamma_{i}\in [0.5,1.0]) \end{array},$$ (13)

$$\mathrm{LSF}3:\;f_{3}{*}^{-1}(\gamma_{i})=\{\begin{array}{l} s_{t-{\left(t^{\varepsilon }-2\times t^{\varepsilon }\times \gamma_{i}\right)}^{1/\varepsilon }},\;(\gamma_{\varepsilon }\in [0,0.5])\\ s_{t+{\left(2\times t^{\beta }\times \gamma_{i}-t^{\beta }\right)}^{1/\beta }},\;(\gamma_{\beta }\in [0.5,1]) \end{array},$$ (14)

Based on the LSF, we propose the operations of IVq-ROULVs.

**Definition 9.** Let $\alpha_{j}=\langle \left[s_{\theta_{j}},s_{\tau_{j}}],(\left[a_{j},b_{j}],[c_{j},d_{j}\right]\right)\rangle (j=1,2,3)$ be any three IVq-ROULVs and *λ* be a real number then

1.
$\alpha_{1}⊕\alpha_{2}=\langle [f{*}^{-1}(f*(\theta_{1})+f*(\theta_{2})-f*(\theta_{1})f*(\theta_{2})),f{*}^{-1}(f*(\tau_{1})+f*(\tau_{2})-f*(\tau_{1})f*(\tau_{2}))],$

  $\left\langle \left(\left[{\left(a_{1}^{q}+a_{2}^{q}-a_{1}^{q}a_{2}^{q}\right)}^{1/q}],[{\left(b_{1}^{q}+b_{2}^{q}-b_{1}^{q}b_{2}^{q}\right)}^{1/q}],[c_{1}c_{2},d_{1}d_{2}\right]\right)\right\rangle$;
2.
$\alpha_{1}⊗\alpha_{2}=\langle [f{*}^{-1}(f*(\theta_{1})\times f*(\theta_{2})),f{*}^{-1}(f*(\tau_{1})\times f*(\tau_{2}))],$

  $\left\langle \left(\left[a_{1}a_{2},b_{1}b_{2}],[{\left(c_{1}^{q}+c_{2}^{q}-c_{1}^{q}c_{2}^{q}\right)}^{1/q}],[{\left(d_{1}^{q}+d_{2}^{q}-d_{1}^{q}d_{2}^{q}\right)}^{1/q}\right]\right)\right\rangle$;
3.
$\lambda \alpha_{3}=\langle [f{*}^{-1}(1-{\left(1-f*(\theta_{3})\right)}^{\lambda }),f{*}^{-1}(1-{\left(1-f*(\tau_{3})\right)}^{\lambda })],$

  $\left(\left[{\left(1-{\left(1-a_{3}^{q}\right)}^{\lambda }\right)}^{1/q},{\left(1-{\left(1-b_{3}^{q}\right)}^{\lambda }\right)}^{1/q}],[c_{3}^{\lambda },c_{4}^{\lambda }\right]\right)\rangle$;
4. $\alpha_{3}^{\lambda }=\langle \left[f{*}^{-1}({\left(f*(\theta_{3})\right)}^{\lambda }),f{*}^{-1}({\left(f*(\tau_{3})\right)}^{\lambda })],(\left[a_{3}^{\lambda },b_{3}^{\lambda }],[{\left(1-{\left(1-c_{3}^{q}\right)}^{\lambda }\right)}^{1/q},{\left(1-{\left(1-d_{3}^{q}\right)}^{\lambda }\right)}^{1/q}\right]\right)\rangle$.

**Theorem 1**. Let *α*_1_, *α*_2_ and *α* be any three IVq-ROULVs, then

1. *α*_1_⊕*α*_2_ = *α*_2_⊕*α*_1_;
2. *α*_1_⊗*α*_2_ = *α*_2_⊗*α*_1_;
3. *λ*(*α*_1_⊕*α*_2_) = *λα*_2_⊕*λα*_1_, *λ*>0;
4. *λ*_1_*α*⊕*λ*_2_*α* = (*λ*_1_+*λ*_2_)*α*, *λ*_1_,*λ*_2_>0;
5. $\alpha_{1}^{\lambda }⊗\alpha_{2}^{\lambda }={\left(\alpha_{2}⊗\alpha_{1}\right)}^{\lambda }$, *λ*>0;
6. $\alpha^{\lambda_{1}}⊗\alpha^{\lambda_{2}}={\left(\alpha \right)}^{\lambda_{1}+\lambda_{2}}$, *λ*_1_,*λ*_2_>0.

**Example 2.** Let *α*_1_ = 〈[*s*_3_,*s*_4_],([0.6,0.7],[0.2,0.3])〉 and *α*_2_ = 〈[*s*_5_,*s*_6_],([0.7,0.8],[0.1,0.2])〉 are two IVq-ROULVs defined on an LTS *S* = {*s*_*i*_|*i* = 0,1,…,6}. Suppose that the absolute semantic gap between any two adjacent linguistic sets is always equal, then LSF1: $f^{*}(s_{i})=\frac{i}{6}(i=0,1,2,\ldots ,6)$ can be applied in the abovementioned operations. According to Definition 9, we can obtain the following results:

1. *α*_1_⊕*α*_2_ = 〈[*s*_5.5_,*s*_6.0_],([0.88,0.94],[0.02,0.06])〉;
2. *α*_1_⊗*α*_2_ = 〈[*s*_2.5_,*s*_4.0_],([0.42,0.56],[0.28,0.44])〉;
3. 2*α*_1_ = 〈[*s*_4.5_,*s*_5.3_],([0.84,0.91],[0.04,0.09])〉;
4. $\alpha_{1}^{2}=\langle \left[s_{1.5},s_{2.7}],(\left[0.36,0.49],[0.36,0.51\right]\right)\rangle$.

### 4.4 Comparison method of IVq-ROULVS

**Definition 10.** Let *α* = 〈[*s*_*θ*_,*s*_*τ*_],([*a*,*b*],[*c*,*d*])〉 be an IVq-ROULV, then the score function of *α* is defined as

$$S(\alpha )=\frac{1}{8}(f^{*}(\theta )+f^{*}(\tau ))(2+a^{q}+b^{q}-c^{q}-d^{q}),$$ (15)

and the accuracy function of *α* is defined as

$$H(\alpha )=\frac{1}{4}(f*(\theta )+f*(\tau ))(a^{q}+b^{q}+c^{q}+d^{q}),$$ (16)

Let $\alpha_{j}=\langle \left[s_{\theta_{j}},s_{\tau_{j}}],(\left[a_{j},b_{j}],[c_{j},d_{j}\right]\right)\rangle (j=1,2)$ be any two IVq-ROULVs, then

1. if *S*(*α*_1_)>*S*(*α*_2_), then *α*_1_>*α*_2_;
2. if *S*(*α*_1_) = *S*(*α*_2_), then if *H*(*α*_1_)>*H*(*α*_2_), then *α*_1_>*α*_2_; if *H*(*α*_1_) = *H*(*α*_2_), then *α*_1_ = *α*_2_.

**Example 3.** Suppose that there are three IVq-ROULVs defined on an LTS *S* = {*s*_*i*_|*i* = 0,1,…,2*t*},

$$\alpha_{1}=\langle \left[s_{2},s_{3}],(\left[0.6,0.7],[0.2,0.3\right]\right)\rangle ;$$

$$\alpha_{2}=\langle \left[s_{1},s_{2}],(\left[0.4,0.5],[0.1,0.2\right]\right)\rangle ;$$

$$\alpha_{3}=\langle \left[s_{2},s_{3}],(\left[0.5,0.8],[0.2,0.3\right]\right)\rangle .$$

Suppose that the absolute deviation between adjacent linguistic subscripts increase with linguistic subscripts *i*(*i* = 0,1,2,…,2*t*;*t* = 3), then LSF2 (*ρ* = 1.37) can be applied in the abovementioned operations. According to Definition 10, we can obtain that (assume that *q* = 1)

$$S(\alpha_{1})=0.3088,S(\alpha_{2})=0.1961,S(\alpha_{3})=0.3088,$$

$$H(\alpha_{1})=0.4191,H(\alpha_{2})=0.2413,H(\alpha_{3})=0.4191.$$

Obviously, we can find that *S*(*α*_1_)>*S*(*α*_2_), then *α*_1_>*α*_2_; that *S*(*α*_1_) = *S*(*α*_3_) and *H*(*α*_1_) = *H*(*α*_3_), then *α*_1_ = *α*_3_.

### 4.5 Distance measure of IVq-ROULVS

**Definition 11.** Let $\alpha_{1}=\langle \left[s_{\theta_{1}},s_{\tau_{1}}],(\left[a_{1},b_{1}],[c_{1},d_{1}\right]\right)\rangle$ and $\alpha_{2}=\langle \left[s_{\theta_{2}},s_{\tau_{2}}],(\left[a_{2},b_{2}],[c_{2},d_{2}\right]\right)\rangle$ be any two IVq-ROULVs, then the Hamming distance between *α*_1_ and *α*_2_ is defined as

$$d(\alpha_{1},\alpha_{2})=\frac{1}{8}(\left|f*(\theta_{1})-f*(\theta_{2})|+|f*(\tau_{1})-f*(\tau_{2})\right|)\times (\left|a_{1}^{q}-a_{2}^{q}|+|b_{1}^{q}-b_{2}^{q}|+|c_{1}^{q}-c_{2}^{q}|+|d_{1}^{q}-d_{2}^{q}\right|),$$ (17)

**Example 4.** Let *α*_1_ = 〈[*s*_3_,*s*_4_],([0.6,0.7],[0.2,0.3])〉 and *α*_2_ = 〈[*s*_5_,*s*_6_],([0.7,0.8],[0.1,0.2])〉 be two IVq-ROULVs (*q* = 2), then we utilize three types of LSFs to calculate the Hamming distance according to Definition 11 and the results can be obtain as follows

$$d_{LSF1}=(\begin{array}{c} \frac{1}{2}(\left|f_{1}*(3)-f_{1}*(4)|+|f_{1}*(5)-f_{1}*(6)\right|)\times \\ \frac{1}{4}(\left|0.6^{2}-0.7^{2}|+|0.7^{2}-0.8^{2}|+|0.2^{2}-0.1^{2}|+|0.3^{2}-0.2^{2}\right|) \end{array})=1\times 0.09=0.09(t=3)$$

Similarly, we can obtain that

$$d_{LSF2}=0.0298(t=3,\rho =1.37)\;\mathrm{and}\;d_{LSF3}=0.0279(t=3,\varepsilon =\beta =0.5).$$

## 5 Some interval-valued q-rung orthopair uncertain linguistic aggregation operators

In this section, we propose some AOs for IVq-ROULVs based on the newly developed operational rules. Properties and special cases of the AOs are also discussed.

### 5.1 The interval-valued q-rung orthopair uncertain linguistic power average operator

**Definition 12.** Let *α*_*i*_(*i* = 1,2,…,*n*) be a series of IVq-ROULVs, then the interval-valued q-rung orthopair uncertain linguistic power average (IVq-ROULPA) operator is given as

$$IVq-ROULPA(\alpha_{1},\alpha_{2},\ldots ,\alpha_{n})=\overset{n}{\underset{i=1}{⊕}}\frac{\left(1+T(\alpha_{i})\right)}{\sum_{i=1}^{n}\left(1+T(\alpha_{i})\right)}\alpha_{i},$$ (18)

where $T(\alpha_{i})=\sum_{j=1;j\neq i}^{n}Sup(\alpha_{i},\alpha_{j})$, *Sup*(*α*_*i*_,*α*_*j*_) denotes the support for *α*_*i*_ from *α*_*j*_, satisfying the conditions

1. *Sup*(*α*_*i*_,*α*_*j*_)∈[0.1]
2. *Sup*(*α*_*i*_,*α*_*j*_) = *Sup*(*α*_*j*_,*α*_*i*_)
3. *Sup*(*α*,*β*)≥*Sup*(*γ*,*ρ*), if *d*(*α*,*β*)≤*d*(*γ*,*ρ*)

To simiplify Eq (18), let

$$\varepsilon_{i}=\frac{1+T(\alpha_{i})}{\sum_{i=1}^{n}\left(1+T(\alpha_{i})\right)},$$ (19)

then, Eq (18) can be written as

$$IVq-ROULPA(\alpha_{1},\alpha_{2},\ldots ,\alpha_{n})=\overset{n}{\underset{i=1}{⊕}}\varepsilon_{i}\alpha_{i},$$ (20)

where $\sum_{i=1}^{n}\varepsilon_{i}=1$ and 0≤*ε*_*i*_≤1.

**Theorem 2.** Let $\alpha_{i}=\langle \left[s_{\theta_{i}},s_{\tau_{i}}],(\left[a_{i},b_{i}],[c_{i},d_{i}\right]\right)\rangle$ (*i* = 1,2,…,*n*) be a series of IVq-ROULVs, then the aggregated value by the IVq-ROULPA operator is still an IVq-ROULV and

$$IVq-ROULPA(\alpha_{1},\alpha_{2},\ldots ,\alpha_{n})=\langle [f{*}^{-1}(1-\prod_{i=1}^{n}{\left(1-f*(\theta_{i})\right)}^{\varepsilon_{i}}),f{*}^{-1}(1-\prod_{i=1}^{n}{\left(1-f*(\tau_{i})\right)}^{\varepsilon_{i}})],$$

$$\langle \left(\left[{\left(1-\prod_{i=1}^{n}{\left(1-a_{i}^{q}\right)}^{\varepsilon_{i}}\right)}^{\frac{1}{q}},{\left(1-\prod_{i=1}^{n}{\left(1-b_{i}^{q}\right)}^{\varepsilon_{i}}\right)}^{\frac{1}{q}}],[\prod_{i=1}^{n}c_{i}^{\varepsilon_{i}},\prod_{i=1}^{n}d_{i}^{\varepsilon_{i}}\right]\right)\rangle .$$ (21)

The proof of Theorem 2 is trivial. In addition, it is easy to prove that the IVq-ROULPA operator has the following properties.

**Theorem 3. (Idempotency)** Let *α*_*i*_ (*i* = 1,2,…,*n*) be a series of IVq-ROULVs, if *α*_*i*_ = *α* = 〈[*s*_*θ*_,*s*_*τ*_], ([*a*,*b*],[*c*,*d*])〉 holds for all *i*, then

$$IVq-ROULPA(\alpha_{1},\alpha_{2},\ldots ,\alpha_{n})=\alpha ,$$ (22)

**Theorem 4. (Boundedness)** Let $\alpha_{i}=\langle \left[s_{\theta_{i}},s_{\tau_{i}}],(\left[a_{i},b_{i}],[c_{i},d_{i}\right]\right)\rangle$ (*i* = 1,2,…,*n*) be a series of IVq-ROULVs, then

$$IVq-ROULPA(\alpha^{-},\alpha^{-},\ldots ,\alpha^{-})\leq IVq-ROULPA(\alpha_{1},\alpha_{2},\ldots ,\alpha_{n})\leq IVq-ROULPA(\alpha^{+},\alpha^{+},\ldots ,\alpha^{+}),$$ (23)

where

$$\alpha^{-}=\langle \left[s_{\overset{n}{\underset{i=1}{\min}}(\theta_{i})},s_{\overset{n}{\underset{i=1}{\min}}(\tau_{i})}],(\left[\overset{n}{\underset{i=1}{\min}}(a_{i}),\overset{n}{\underset{i=1}{\min}}(b_{i})],[\overset{n}{\underset{i=1}{\max}}(c_{i}),\overset{n}{\underset{i=1}{\max}}(d_{i})\right]\right)\rangle ,$$

and

$$\alpha^{+}=\langle \left[s_{\overset{n}{\underset{i=1}{\max}}(\theta_{i})},s_{\overset{n}{\underset{i=1}{\max}}(\tau_{i})}],(\left[\overset{n}{\underset{i=1}{\max}}(a_{i}),\overset{n}{\underset{i=1}{\max}}(b_{i})],[\overset{n}{\underset{i=1}{\min}}(c_{i}),\overset{n}{\underset{i=1}{\min}}(d_{i})\right]\right)\rangle ,$$

### 5.2 The interval-valued q-rung orthopair uncertain linguistic power weighted average operator

**Definition 13.** Let *α*_*i*_(*i* = 1,2,…,*n*) be a series of IVq-ROULVs and *w* = (*w*_1_,*w*_2_,…,*w*_*n*_]^*T*^ be the corresponding weight vector, satisfying 0≤*w*_*i*_≤1 and $\sum_{i=1}^{n}w_{i}=1$. Then the interval-valued q-rung orthopair uncertain linguistic power weighted average (IVq-ROULPWA) operator is expressed as

$$IVq-ROULPWA(\alpha_{1},\alpha_{2},\ldots ,\alpha_{n})=\overset{n}{\underset{i=1}{⊕}}\frac{w_{i}(1+T(\alpha_{i}))}{\sum_{i=1}^{n}w_{i}(1+T(\alpha_{i}))}\alpha_{i},$$ (24)

where $T(\alpha_{i})=\sum_{j=1;j\neq i}^{n}Sup(\alpha_{i},\alpha_{j})$, *Sup*(*α*_*i*_,*α*_*j*_) denotes the support for *a*_*i*_ from *a*_*j*_, satisfying the conditions presented in Definition 12.

To simiplify Eq (24), let

$$\xi_{i}=\frac{w_{i}(1+T(\alpha_{i}))}{\sum_{i=1}^{n}w_{i}(1+T(\alpha_{i}))},$$ (25)

then, Eq (24) can be written as

$$IVq-ROULPWA(\alpha_{1},\alpha_{2},\ldots ,\alpha_{n})=\overset{n}{\underset{i=1}{⊕}}\xi_{i}\alpha_{i},$$ (26)

where $\sum_{i=1}^{n}\xi_{i}=1$ and 0≤*ξ*_*i*_≤1.

**Theorem 5.** Let $\alpha_{i}=\langle \left[s_{\theta_{i}},s_{\tau_{i}}],(\left[a_{i},b_{i}],[c_{i},d_{i}\right]\right)\rangle$ (*i* = 1,2,…,*n*) be a series of IVq-ROULVs, then the aggregated value by the IVq-ROULPWA operator is still an IVq-ROULV and

$$IVq-ROULPWA(\alpha_{1},\alpha_{2},\ldots ,\alpha_{n})=\langle [f{*}^{-1}(1-\prod_{i=1}^{n}{\left(1-f*(\theta_{i})\right)}^{\xi_{i}}),f{*}^{-1}(1-\prod_{i=1}^{n}{\left(1-f*(\tau_{i})\right)}^{\xi_{i}})],$$

$$\left(\left[{\left(1-\prod_{i=1}^{n}{\left(1-a_{i}^{q}\right)}^{\xi_{i}}\right)}^{\frac{1}{q}},{\left(1-\prod_{i=1}^{n}{\left(1-b_{i}^{q}\right)}^{\xi_{i}}\right)}^{\frac{1}{q}}],[\prod_{i=1}^{n}c_{i}^{\xi_{i}},\prod_{i=1}^{n}d_{i}^{\xi_{i}}\right]\right)\rangle ,$$ (27)

### 5.3 The interval-valued q-rung orthopair uncertain linguistic power Muirhead mean operator

**Definition 14**. Let *α*_*j*_(*j* = 1,2,…,*n*) be a series of IVq-ROULVs, and *H* = (*h*_1_,*h*_2_,…,*h*_*n*_)∈*R*^*n*^ be a set of parameters. The interval-valued q-rung orthopair uncertain linguistic power Muirhead mean (IVq-ROULPMM) operator is expressed as

$$IVq-ROULPMM^{H}(\alpha_{1},\alpha_{2},\ldots ,\alpha_{n})={\left(\frac{1}{n!}\underset{\zeta \in T_{n}}{⊕}\overset{n}{\underset{j=1}{⊗}}{\left(n\frac{\left(1+T(\alpha_{\zeta (j)})\right)}{\sum_{j=1}^{n}\left(1+T(\alpha_{j})\right)}\alpha_{\zeta (j)}\right)}^{h_{j}}\right)}^{\frac{1}{\sum_{j=1}^{n}h_{j}}},$$ (28)

where

$$T(\alpha_{j})=\sum_{i=1.j\neq i}^{n}Sup(\alpha_{i},\alpha_{j}),$$ (29)

and

$$Sup(\alpha_{i},\alpha_{j})=1-d(\alpha_{i},\alpha_{j}),$$ (30)

*ζ*(*j*)(*j* = 1,2,…,*n*) denotes any permutation of (1,2,…,*n*), *T*_*n*_ represents all possible permutations of (1,2,…,*n*), and *n* is the balancing coefficient. *d*(*α*_*i*_,*α*_*j*_) represents the Hamming distance between *α*_*i*_ and *α*_*j*_, and *Sup*(*α*_*i*_,*α*_*j*_) is the support for *α*_*i*_ from *α*_*j*_, satisfying the properties presented in Definition 12.

To simiplify Eq (30), let

$$\varpi_{j}=\frac{1+T(\alpha_{j})}{\sum_{j=1}^{n}\left(1+T(\alpha_{j})\right)},$$ (31)

then, Eq (30) can be written as

$$IVq-ROULPMM^{H}(\alpha_{1},\alpha_{2},\ldots ,\alpha_{n})={\left(\frac{1}{n!}\underset{\varsigma \in T_{n}}{⊕}\overset{n}{\underset{j=1}{⊗}}{\left(n\varpi_{\varsigma (j)}\alpha_{\varsigma (j)}\right)}^{h_{j}}\right)}^{\frac{1}{\sum_{j=1}^{n}h_{j}}},$$ (32)

where $\sum_{j=1}^{n}\varpi_{j}=1$ and 0≤*ϖ*_*j*_≤1.

According to Definition 9, the following theorem can be obtained.

**Theorem 6.** Let $\alpha_{j}=\langle \left[s_{\theta_{j}},s_{\tau_{j}}],(\left[a_{j},b_{j}],[c_{j},d_{j}\right]\right)\rangle$ (*j* = 1,2,…,*n*) be a series of IVq-ROULVs, the aggregated value by the IVq-ROULPMM operator is still an IVq-ROULV and

$$IVq-ROULPMM^{H}(\alpha_{1},\alpha_{2},\ldots ,\alpha_{n})=\langle [f{*}^{-1}({\left(1-\prod_{\varsigma \in T_{n}}{\left(1-\prod_{j=1}^{n}{\left(1-{\left(1-f*(\theta_{\varsigma (j)})\right)}^{n\varpi_{\varsigma (j)}}\right)}^{h_{j}}\right)}^{\frac{1}{n!}}\right)}^{\frac{1}{\sum_{j=1}^{n}h_{j}}}),$$

$$f{*}^{-1}({\left(1-\prod_{\varsigma \in T_{n}}{\left(1-\prod_{j=1}^{n}{\left(1-{\left(1-f*(\tau_{\varsigma (j)})\right)}^{n\varpi_{\varsigma (j)}}\right)}^{h_{j}}\right)}^{\frac{1}{n!}}\right)}^{\frac{1}{\sum_{j=1}^{n}h_{j}}})],$$

$$(\left[{\left(1-\prod_{\varsigma \in T_{n}}{\left(1-\prod_{j=1}^{n}{\left(1-{\left(1-a_{\varsigma (j)}^{q}\right)}^{n\varpi_{\varsigma (j)}}\right)}^{h_{j}}\right)}^{\frac{1}{n!}}\right)}^{\frac{1}{q\sum_{j=1}^{n}h_{j}}},{\left(1-\prod_{\varsigma \in T_{n}}{\left(1-\prod_{j=1}^{n}{\left(1-{\left(1-b_{\varsigma (j)}^{q}\right)}^{n\varpi_{\varsigma (j)}}\right)}^{h_{j}}\right)}^{\frac{1}{n!}}\right)}^{\frac{1}{q\sum_{j=1}^{n}h_{j}}}\right],$$

$$\left[{\left(1-{\left(1-\prod_{\varsigma \in T_{n}}{\left(1-\prod_{j=1}^{n}{\left(1-{\left(c_{\varsigma (j)}^{n\varpi_{\varsigma (j)}}\right)}^{q}\right)}^{h_{j}}\right)}^{\frac{1}{n!}}\right)}^{\frac{1}{\sum_{j=1}^{n}h_{j}}}\right)}^{\frac{1}{q}},{\left(1-{\left(1-\prod_{\varsigma \in T_{n}}{\left(1-\prod_{j=1}^{n}{\left(1-{\left(d_{\varsigma (j)}^{n\varpi_{\varsigma (j)}}\right)}^{q}\right)}^{h_{j}}\right)}^{\frac{1}{n!}}\right)}^{\frac{1}{\sum_{j=1}^{n}h_{j}}}\right)}^{\frac{1}{q}}\right]\rangle$$ (33)

***Proof*.** According to Definition 9, we can get

$$n\varpi_{\varsigma (j)}\alpha_{\varsigma (j)}=\langle [f{*}^{-1}(1-{\left(1-f*(\theta_{\varsigma (j)})\right)}^{n\varpi_{\varsigma (j)}}),f{*}^{-1}(1-{\left(1-f*(\tau_{\varsigma (j)})\right)}^{n\varpi_{\varsigma (j)}})],$$

$$\left(\left[{\left(1-{\left(1-a_{\varsigma (j)}^{q}\right)}^{n\varpi_{\varsigma (j)}}\right)}^{\frac{1}{q}},{\left(1-{\left(1-b_{\varsigma (j)}^{q}\right)}^{n\varpi_{\varsigma (j)}}\right)}^{\frac{1}{q}}],[c_{\varsigma (j)}^{n\varpi_{\varsigma (j)}},d_{\varsigma (j)}^{n\varpi_{\varsigma (j)}}\right]\right)\rangle .$$

and

$${\left(n\varpi_{\varsigma (j)}\alpha_{\varsigma (j)}\right)}^{h_{j}}=\langle [f{*}^{-1}({\left(1-{\left(1-f*(\theta_{\varsigma (j)})\right)}^{n\varpi_{\varsigma (j)}}\right)}^{h_{j}}),f{*}^{-1}({\left(1-{\left(1-f*(\tau_{\varsigma (j)})\right)}^{n\varpi_{\varsigma (j)}}\right)}^{h_{j}})],$$

$$\left(\left[{\left(1-{\left(1-a_{\varsigma (j)}^{q}\right)}^{n\varpi_{\varsigma (j)}}\right)}^{\frac{1}{q}}{}^{h_{j}},{\left(1-{\left(1-b_{\varsigma (j)}^{q}\right)}^{n\varpi_{\varsigma (j)}}\right)}^{\frac{1}{q}}{}^{h_{j}}],[{\left(1-{\left(1-{\left(c_{\varsigma (j)}^{n\varpi_{\varsigma (j)}}\right)}^{q}\right)}^{h_{j}}\right)}^{\frac{1}{q}},{\left(1-{\left(1-{\left(d_{\varsigma (j)}^{n\varpi_{\varsigma (j)}}\right)}^{q}\right)}^{h_{j}}\right)}^{\frac{1}{q}}\right]\right)\rangle$$

Therefore,

$$\overset{n}{\underset{j=1}{⊗}}{\left(n\varpi_{\varsigma (j)}\alpha_{\varsigma (j)}\right)}^{h_{j}}=\langle [f{*}^{-1}(\prod_{j=1}^{n}{\left(1-{\left(1-f*(\theta_{\varsigma (j)})\right)}^{n\varpi_{\varsigma (j)}}\right)}^{h_{j}}),f{*}^{-1}(\prod_{j=1}^{n}{\left(1-{\left(1-f*(\tau_{\varsigma (j)})\right)}^{n\varpi_{\varsigma (j)}}\right)}^{h_{j}})],$$

$$([\prod_{j=1}^{n}{\left(1-{\left(1-a_{\varsigma (j)}^{q}\right)}^{n\varpi_{\varsigma (j)}}\right)}^{\frac{1}{q}}{}^{h_{j}},\prod_{j=1}^{n}{\left(1-{\left(1-b_{\varsigma (j)}^{q}\right)}^{n\varpi_{\varsigma (j)}}\right)}^{\frac{1}{q}}{}^{h_{j}}],$$

$$\left[{\left(1-\prod_{j=1}^{n}{\left(1-{\left(c_{\varsigma (j)}^{n\varpi_{\varsigma (j)}}\right)}^{q}\right)}^{h_{j}}\right)}^{\frac{1}{q}},{\left(1-\prod_{j=1}^{n}{\left(1-{\left(d_{\varsigma (j)}^{n\varpi_{\varsigma (j)}}\right)}^{q}\right)}^{h_{j}}\right)}^{\frac{1}{q}}\right])\rangle .$$

Further,

$$\underset{\zeta \in T_{n}}{⊕}\overset{n}{\underset{j=1}{⊗}}{\left(n\varpi_{\varsigma (j)}\alpha_{\varsigma (j)}\right)}^{h_{j}}=\langle [f{*}^{-1}(1-\prod_{\varsigma \in T_{n}}\left(1-\prod_{j=1}^{n}{\left(1-{\left(1-f*(\theta_{\varsigma (j)})\right)}^{n\varpi_{\varsigma (j)}}\right)}^{h_{j}}\right)),$$

$$f{*}^{-1}(1-\prod_{\varsigma \in T_{n}}\left(1-\prod_{j=1}^{n}{\left(1-{\left(1-f*(\tau_{\varsigma (j)})\right)}^{n\varpi_{\varsigma (j)}}\right)}^{h_{j}}\right)),$$

$$([{\left(1-\prod_{\varsigma \in T_{n}}\left(1-\prod_{j=1}^{n}{\left(1-{\left(1-a_{\varsigma (j)}^{q}\right)}^{n\varpi_{\varsigma (j)}}\right)}^{h_{j}}\right)\right)}^{1/q},{\left(1-\prod_{\varsigma \in T_{n}}\left(1-\prod_{j=1}^{n}{\left(1-{\left(1-b_{\varsigma (j)}^{q}\right)}^{n\varpi_{\varsigma (j)}}\right)}^{h_{j}}\right)\right)}^{1/q}],$$

$$\left(\left[\prod_{\varsigma \in T_{n}}{\left(1-\prod_{j=1}^{n}{\left(1-{\left(c_{\varsigma (j)}^{n\varpi_{\varsigma (j)}}\right)}^{q}\right)}^{h_{j}}\right)}^{1/q},\prod_{\varsigma \in T_{n}}{\left(1-\prod_{j=1}^{n}{\left(1-{\left(d_{\varsigma (j)}^{n\varpi_{\varsigma (j)}}\right)}^{q}\right)}^{h_{j}}\right)}^{1/q}\right]\right)\rangle .$$

Then,

$$\frac{1}{n!}\underset{\zeta \in T_{n}}{⊕}\overset{n}{\underset{j=1}{⊗}}{\left(n\varpi_{\varsigma (j)}\alpha_{\varsigma (j)}\right)}^{h_{j}}=\langle [f{*}^{-1}(1-\prod_{\varsigma \in T_{n}}{\left(1-\prod_{j=1}^{n}{\left(1-{\left(1-f*(\theta_{\varsigma (j)})\right)}^{n\varpi_{\varsigma (j)}}\right)}^{h_{j}}\right)}^{\frac{1}{n!}}),$$

$$f{*}^{-1}(1-\prod_{\varsigma \in T_{n}}{\left(1-\prod_{j=1}^{n}{\left(1-{\left(1-f*(\tau_{\varsigma (j)})\right)}^{n\varpi_{\varsigma (j)}}\right)}^{h_{j}}\right)}^{\frac{1}{n!}})],$$

$$([{\left(1-{\left(\prod_{\varsigma \in T_{n}}\left(1-\prod_{j=1}^{n}{\left(1-{\left(1-a_{\varsigma (j)}^{q}\right)}^{n\varpi_{\varsigma (j)}}\right)}^{h_{j}}\right)\right)}^{\frac{1}{n!}}\right)}^{1/q},{\left(1-{\left(\prod_{\varsigma \in T_{n}}\left(1-\prod_{j=1}^{n}{\left(1-{\left(1-b_{\varsigma (j)}^{q}\right)}^{n\varpi_{\varsigma (j)}}\right)}^{h_{j}}\right)\right)}^{\frac{1}{n!}}\right)}^{1/q}],$$

$$\left[{\left(\prod_{\varsigma \in T_{n}}{\left(1-\prod_{j=1}^{n}{\left(1-{\left(c_{\varsigma (j)}^{n\varpi_{\varsigma (j)}}\right)}^{q}\right)}^{h_{j}}\right)}^{1/q}\right)}^{\frac{1}{n!}},{\left(\prod_{\varsigma \in T_{n}}{\left(1-\prod_{j=1}^{n}{\left(1-{\left(d_{\varsigma (j)}^{n\varpi_{\varsigma (j)}}\right)}^{q}\right)}^{h_{j}}\right)}^{1/q}\right)}^{\frac{1}{n!}}\right]\rangle .$$

Thus,

$${\left(\frac{1}{n!}\underset{\varsigma \in T_{n}}{⊕}\overset{n}{\underset{j=1}{⊗}}{\left(n\varpi_{\varsigma (j)}\alpha_{\varsigma (j)}\right)}^{h_{j}}\right)}^{\frac{1}{\sum_{j=1}^{n}h_{j}}}=\langle [f{*}^{-1}({\left(1-\prod_{\varsigma \in T_{n}}{\left(1-\prod_{j=1}^{n}{\left(1-{\left(1-f*(\theta_{\varsigma (j)})\right)}^{n\varpi_{\varsigma (j)}}\right)}^{h_{j}}\right)}^{\frac{1}{n!}}\right)}^{\frac{1}{\sum_{j=1}^{n}h_{j}}}),$$

$$f{*}^{-1}({\left(1-\prod_{\varsigma \in T_{n}}{\left(1-\prod_{j=1}^{n}{\left(1-{\left(1-f*(\tau_{\varsigma (j)})\right)}^{n\varpi_{\varsigma (j)}}\right)}^{h_{j}}\right)}^{\frac{1}{n!}}\right)}^{\frac{1}{\sum_{j=1}^{n}h_{j}}})],$$

$$(\left[{\left(1-\prod_{\varsigma \in T_{n}}{\left(1-\prod_{j=1}^{n}{\left(1-{\left(1-a_{\varsigma (j)}^{q}\right)}^{n\varpi_{\varsigma (j)}}\right)}^{h_{j}}\right)}^{\frac{1}{n!}}\right)}^{\frac{1}{q\sum_{j=1}^{n}h_{j}}},{\left(1-\prod_{\varsigma \in T_{n}}{\left(1-\prod_{j=1}^{n}{\left(1-{\left(1-b_{\varsigma (j)}^{q}\right)}^{n\varpi_{\varsigma (j)}}\right)}^{h_{j}}\right)}^{\frac{1}{n!}}\right)}^{\frac{1}{q\sum_{j=1}^{n}h_{j}}}\right],$$

$$\left[{\left(1-{\left(1-{\left(\prod_{\varsigma \in T_{n}}\left(1-\prod_{j=1}^{n}{\left(1-{\left(c_{\varsigma (j)}^{n\varpi_{\varsigma (j)}}\right)}^{q}\right)}^{h_{j}}\right)\right)}^{\frac{1}{n!}}\right)}^{\frac{1}{\sum_{j=1}^{n}h_{j}}}\right)}^{\frac{1}{q}},{\left(1-{\left(1-{\left(\prod_{\varsigma \in T_{n}}\left(1-\prod_{j=1}^{n}{\left(1-{\left(d_{\varsigma (j)}^{n\varpi_{\varsigma (j)}}\right)}^{q}\right)}^{h_{j}}\right)\right)}^{\frac{1}{n!}}\right)}^{\frac{1}{\sum_{j=1}^{n}h_{j}}}\right)}^{\frac{1}{q}}\right])\rangle .$$

**Theorem 7. (Idempotency)** Let $\alpha_{j}=\langle \left[s_{\theta_{j}},s_{\tau_{j}}],(\left[a_{j},b_{j}],[c_{j},d_{j}\right]\right)\rangle$ (*j* = 1,2,…,*n*) be a series of IVq-ROULVs, if *α*_*j*_ = *α* = 〈[*s*_*θ*_,*s*_*τ*_],([*a*,*b*],[*c*,*d*])〉 holds for all *j*, then

$$IVq-ROULPMM^{H}(\alpha_{1},\alpha_{2},\ldots ,\alpha_{n})=\alpha ,$$ (34)

***Proof*.** Since *α* = 〈[*s*_*θ*_,*s*_*τ*_],([*a*,*b*],[*c*,*d*])〉 (*j* = 1,2,…,*n*), we can get *Sup*(*α*_*i*_,*α*_*j*_) = 1/*n* for *i*,*j* = 1,2,…,*n* and *i*≠*j*. Thus, we can derive *ϖ*_*j*_ = 1/*n*(*j* = 1,2,…,*n*). According to Theorem 6, we have

$${\left(\frac{1}{n!}\underset{\varsigma \in T_{n}}{⊕}\overset{n}{\underset{j=1}{⊗}}{\left(n\varpi_{\varsigma (j)}\alpha_{\varsigma (j)}\right)}^{h_{j}}\right)}^{\frac{1}{\sum_{j=1}^{n}h_{j}}}=\langle [f{*}^{-1}({\left(1-\prod_{\varsigma \in T_{n}}{\left(1-\prod_{j=1}^{n}{\left(1-(1-f*(\theta ))\right)}^{h_{j}}\right)}^{\frac{1}{n!}}\right)}^{\frac{1}{\sum_{j=1}^{n}h_{j}}}),$$

$$f{*}^{-1}({\left(1-\prod_{\varsigma \in T_{n}}{\left(1-\prod_{j=1}^{n}{\left(1-(1-f*(\tau ))\right)}^{h_{j}}\right)}^{\frac{1}{n!}}\right)}^{\frac{1}{\sum_{j=1}^{n}h_{j}}})],$$

$$([{\left(1-\prod_{\varsigma \in T_{n}}{\left(1-\prod_{j=1}^{n}{\left(1-(1-a^{q})\right)}^{h_{j}}\right)}^{\frac{1}{n!}}\right)}^{\frac{1}{q\sum_{j=1}^{n}h_{j}}},{\left(1-\prod_{\varsigma \in T_{n}}{\left(1-\prod_{j=1}^{n}{\left(1-(1-b^{q})\right)}^{h_{j}}\right)}^{\frac{1}{n!}}\right)}^{\frac{1}{q\sum_{j=1}^{n}h_{j}}}],$$

$$\left[{\left(1-{\left(1-{\left(\prod_{\varsigma \in T_{n}}\left(1-\prod_{j=1}^{n}{\left(1-{\left(c\right)}^{q}\right)}^{h_{j}}\right)\right)}^{\frac{1}{n!}}\right)}^{\frac{1}{\sum_{j=1}^{n}h_{j}}}\right)}^{1/q},{\left(1-{\left(1-{\left(\prod_{\varsigma \in T_{n}}\left(1-\prod_{j=1}^{n}{\left(1-{\left(d\right)}^{q}\right)}^{h_{j}}\right)\right)}^{\frac{1}{n!}}\right)}^{\frac{1}{\sum_{j=1}^{n}h_{j}}}\right)}^{1/q}\right])\rangle$$

=⟨[θ,τ],([a,b],[c,d])⟩=α.

**Theorem 8. (Boundedness)** Let $\alpha_{j}=\langle \left[s_{\theta_{j}},s_{\tau_{j}}],(\left[a_{j},b_{j}],[c_{j},d_{j}\right]\right)\rangle$ (*j* = 1,2,…,*n*) be a series of IVq-ROULVs, then

$$\alpha^{-}\leq IVq-ROULPMM^{H}(\alpha_{1},\alpha_{2},\ldots ,\alpha_{n})\leq \alpha^{+},$$ (35)

where

$$\alpha^{-}=\langle \left[s_{\overset{n}{\underset{j=1}{\min}}(\theta_{j})},s_{\overset{n}{\underset{j=1}{\min}}(\tau_{j})}],(\left[\overset{n}{\underset{j=1}{\min}}(a_{j}),\overset{n}{\underset{j=1}{\min}}(b_{j})],[\overset{n}{\underset{j=1}{\max}}(c_{j}),\overset{n}{\underset{j=1}{\max}}(d_{j})\right]\right)\rangle ,$$

and

$$\alpha^{+}=\langle \left[s_{\overset{n}{\underset{j=1}{\max}}(\theta_{j})},s_{\overset{n}{\underset{j=1}{\max}}(\tau_{j})}],(\left[\overset{n}{\underset{j=1}{\max}}(a_{j}),\overset{n}{\underset{j=1}{\max}}(b_{j})],[\overset{n}{\underset{j=1}{\min}}(c_{j}),\overset{n}{\underset{j=1}{\min}}(d_{j})\right]\right)\rangle .$$

***Proof*.** According to Theorems 6 and 7, it is easy to obtain that

$$f{*}^{-1}({\left(1-\prod_{\varsigma \in T_{n}}{\left(1-\prod_{j=1}^{n}{\left(1-{\left(1-f*(\theta_{\varsigma (j)})\right)}^{n\varpi_{\varsigma (j)}}\right)}^{h_{j}}\right)}^{\frac{1}{n!}}\right)}^{\frac{1}{\sum_{j=1}^{n}h_{j}}})\geq$$

$$f{*}^{-1}({\left(1-\prod_{\varsigma \in T_{n}}{\left(1-\prod_{j=1}^{n}{\left(1-{\left(1-f*(\overset{n}{\underset{j=1}{\min}}\theta_{\varsigma (j)})\right)}^{n\varpi_{\varsigma (j)}}\right)}^{h_{j}}\right)}^{\frac{1}{n!}}\right)}^{\frac{1}{\sum_{j=1}^{n}h_{j}}})=\overset{n}{\underset{j=1}{\min}}(\theta_{j}).$$

Similarly, we have

$$f{*}^{-1}({\left(1-\prod_{\varsigma \in T_{n}}{\left(1-\prod_{j=1}^{n}{\left(1-{\left(1-f*(\tau_{\varsigma (j)})\right)}^{n\varpi_{\varsigma (j)}}\right)}^{h_{j}}\right)}^{\frac{1}{n!}}\right)}^{\frac{1}{\sum_{j=1}^{n}h_{j}}})\geq \overset{n}{\underset{j=1}{\min}}(\tau_{j}),$$

$${\left(1-\prod_{\varsigma \in T_{n}}{\left(1-\prod_{j=1}^{n}{\left(1-{\left(1-a_{\varsigma (j)}^{q}\right)}^{n\varpi_{\varsigma (j)}}\right)}^{h_{j}}\right)}^{\frac{1}{n!}}\right)}^{\frac{1}{q\sum_{j=1}^{n}h_{j}}}\geq \overset{n}{\underset{j=1}{\min}}(a_{j}),$$

$${\left(1-\prod_{\varsigma \in T_{n}}{\left(1-\prod_{j=1}^{n}{\left(1-{\left(1-b_{\varsigma (j)}^{q}\right)}^{n\varpi_{\varsigma (j)}}\right)}^{h_{j}}\right)}^{\frac{1}{n!}}\right)}^{\frac{1}{q\sum_{j=1}^{n}h_{j}}}\geq \overset{n}{\underset{j=1}{\min}}(b_{j}),$$

$${\left(1-{\left(1-{\left(\prod_{\varsigma \in T_{n}}\left(1-\prod_{j=1}^{n}{\left(1-{\left(c_{\varsigma (j)}^{n\varpi_{\varsigma (j)}}\right)}^{q}\right)}^{h_{j}}\right)\right)}^{\frac{1}{n!}}\right)}^{\frac{1}{\sum_{j=1}^{n}h_{j}}}\right)}^{\frac{1}{q}}\leq \overset{n}{\underset{j=1}{\max}}(c_{j}),$$

$${\left(1-{\left(1-{\left(\prod_{\varsigma \in T_{n}}\left(1-\prod_{j=1}^{n}{\left(1-{\left(d_{\varsigma (j)}^{n\varpi_{\varsigma (j)}}\right)}^{q}\right)}^{h_{j}}\right)\right)}^{\frac{1}{n!}}\right)}^{\frac{1}{\sum_{j=1}^{n}h_{j}}}\right)}^{\frac{1}{q}}\leq \overset{n}{\underset{j=1}{\max}}(d_{j}).$$

According to Definition 10, we can get *α*^−^≤*IVq*−*ROULPMM*^*H*^ (*α*_1_,*α*_2_,…,*α*_n_). Similarly, we have *IVq*−*ROULPMM*^*H*^ (*α*_1_,*α*_2_,…,*α*_n_)≤*α*^+^ and thus *α*^−^≤*IVq*−*ROULPMM*^*H*^ (*α*_1_,*α*_2_,…,*α*_n_)≤*α*^+^.

In the followings, we discuss some special cases of the IVq-ROULPMM operator with respect to *H* and *q*.

**Special case 1**: if *H* = (1,0,…,0), then the IVq-ROULPMM operator reduces to the IVq-ROULPA operator, i.e.


$$IVq-ROULPMM^{\left(1,0,\ldots ,0\right)}(\alpha_{1},\alpha_{2},\ldots ,\alpha_{n})=\langle [f{*}^{-1}(1-\prod_{j=1}^{n}{\left(1-f*(\theta_{j})\right)}^{\varpi_{j}}),f{*}^{-1}(1-\prod_{j=1}^{n}{\left(1-f*(\tau_{j})\right)}^{\varpi_{j}})],$$

$$\left(\left[{\left(1-\prod_{j=1}^{n}{\left(1-a_{j}^{q}\right)}^{\varpi_{j}}\right)}^{\frac{1}{q}},{\left(1-\prod_{j=1}^{n}{\left(1-b_{j}^{q}\right)}^{\varpi_{j}}\right)}^{\frac{1}{q}}],[\prod_{j=1}^{n}c_{j}^{\varpi_{j}},\prod_{j=1}^{n}d_{j}^{\varpi_{j}}\right]\right)\rangle =\overset{n}{\underset{j=1}{⊕}}\varpi_{j}\alpha_{j}.$$ (36)

In this case, if *Sup*(*α*_*i*_,*α*_*j*_) = *t*>0 for all *i*≠*j*, then the IVq-ROULPMM operator reduces to the interval-valued q-rung orthopair uncertain linguistic average (IVq-ROULA) operator, i.e.,

$$IVq-ROULPMM^{\left(1,0,\ldots ,0\right)}(\alpha_{1},\alpha_{2},\ldots ,\alpha_{n})=\langle [f{*}^{-1}(1-\prod_{j=1}^{n}{\left(1-f*(\theta_{j})\right)}^{\frac{1}{n}}),f{*}^{-1}(1-\prod_{j=1}^{n}{\left(1-f*(\tau_{j})\right)}^{\frac{1}{n}})],$$

$$\langle \left(\left[{\left(1-\prod_{j=1}^{n}{\left(1-a_{j}^{q}\right)}^{\frac{1}{n}}\right)}^{\frac{1}{q}},{\left(1-\prod_{j=1}^{n}{\left(1-b_{j}^{q}\right)}^{\frac{1}{n}}\right)}^{\frac{1}{q}}],[\prod_{j=1}^{n}c_{j}^{\frac{1}{n}},\prod_{j=1}^{n}d_{j}^{\frac{1}{n}}\right]\right)\rangle =\frac{1}{n}\overset{n}{\underset{j=1}{⊕}}\alpha_{j}.$$ (37)

**Special case 2**: if *H* =(1,1,0,…,0), then the IVq-ROULPMM operator reduces to the interval-valued q-rung orthopair uncertain linguistic power Bonferroni mean (IVq-ROULPBM) operator, i.e.,

$$IVq-ROULPMM^{\left(1,1,0,\ldots ,0\right)}(\alpha_{1},\alpha_{2},\ldots ,\alpha_{n})=$$

$$\langle [f{*}^{-1}({\left(1-\prod_{\begin{array}{l} i,j=1\\ i\neq j \end{array}}^{n}{\left(1-(1-{\left(1-f*(\theta_{i})\right)}^{n\varpi_{i}})\times (1-{\left(1-f*(\theta_{j})\right)}^{n\varpi_{j}})\right)}^{\frac{1}{n(n-1)}}\right)}^{\frac{1}{2}}),$$

$$f{*}^{-1}({\left(1-\prod_{\begin{array}{l} i,j=1\\ i\neq j \end{array}}^{n}{\left(1-(1-{\left(1-f*(\tau_{i})\right)}^{n\varpi_{i}})\times (1-{\left(1-f*(\tau_{j})\right)}^{n\varpi_{j}})\right)}^{\frac{1}{n(n-1)}}\right)}^{\frac{1}{2}})],$$

$$(\left[{\left(1-\prod_{\begin{array}{l} i,j=1\\ i\neq j \end{array}}^{n}{\left(1-(1-{\left(1-a_{i}^{q}\right)}^{n\varpi_{i}})(1-{\left(1-a_{j}^{q}\right)}^{n\varpi_{j}})\right)}^{\frac{1}{n(n-1)}}\right)}^{\frac{1}{2q}},{\left(1-\prod_{\begin{array}{l} i,j=1\\ i\neq j \end{array}}^{n}{\left(1-(1-{\left(1-b_{i}^{q}\right)}^{n\varpi_{i}})(1-{\left(1-b_{j}^{q}\right)}^{n\varpi_{j}})\right)}^{\frac{1}{n(n-1)}}\right)}^{\frac{1}{2q}}\right],$$

$$[{\left(1-{\left(1-\prod_{\begin{array}{l} i,j=1\\ i\neq j \end{array}}^{n}{\left({\left(c_{i}^{n\varpi_{i}}\right)}^{q}+{\left(c_{j}^{n\varpi_{i}}\right)}^{q}-{\left(c_{i}^{n\varpi_{i}}\right)}^{q}{\left(c_{j}^{n\varpi_{i}}\right)}^{q}\right)}^{\frac{1}{n(n-1)}}\right)}^{\frac{1}{2}}\right)}^{1/q},$$

$${\left(1-{\left(1-\prod_{\begin{array}{l} i,j=1\\ i\neq j \end{array}}^{n}{\left({\left(d_{i}^{n\varpi_{i}}\right)}^{q}+{\left(d_{j}^{n\varpi_{i}}\right)}^{q}-{\left(d_{i}^{n\varpi_{i}}\right)}^{q}{\left(d_{j}^{n\varpi_{i}}\right)}^{q}\right)}^{\frac{1}{n(n-1)}}\right)}^{\frac{1}{2}}\right)}^{1/q}])\rangle$$

$$={\left(\frac{1}{n(n-1)}\overset{n}{\underset{\begin{array}{l} i,j=1\\ i\neq j \end{array}}{⊕}}(n\varpi_{i}\alpha_{i}⊗n\varpi_{j}\alpha_{j})\right)}^{\frac{1}{2}}.$$ (38)

In this case, if *Sup*(*α*_*i*_,*α*_*j*_) = *t*>0 for all *i*≠*j*, then the IVq-ROULPMM operator reduces to the interval-valued q-rung orthopair uncertain linguistic Bonferroni mean (IVq-ROULBM) operator, i.e.,

$$IVq-ROULPMM^{\left(1,1,0,\ldots ,0\right)}(\alpha_{1},\alpha_{2},\ldots ,\alpha_{n})=$$

$$\langle [f{*}^{-1}({\left(1-\prod_{\begin{array}{l} i,j=1\\ i\neq j \end{array}}^{n}{\left(1-f*(\theta_{i})f*(\theta_{j})\right)}^{\frac{1}{n(n-1)}}\right)}^{\frac{1}{2}}),f{*}^{-1}({\left(1-\prod_{\begin{array}{l} i,j=1\\ i\neq j \end{array}}^{n}{\left(1-f*(\tau_{i})f*(\tau_{j})\right)}^{\frac{1}{n(n-1)}}\right)}^{\frac{1}{2}})],$$

$$([{\left(1-\prod_{\begin{array}{l} i,j=1\\ i\neq j \end{array}}^{n}{\left(1-a_{i}^{q}a_{j}^{q}\right)}^{\frac{1}{n(n-1)}}\right)}^{\frac{1}{2q}},{\left(1-\prod_{\begin{array}{l} i,j=1\\ i\neq j \end{array}}^{n}{\left(1-b_{i}^{q}b_{j}^{q}\right)}^{\frac{1}{n(n-1)}}\right)}^{\frac{1}{2q}}],$$

$$[{\left(1-{\left(1-\prod_{\begin{array}{l} i,j=1\\ i\neq j \end{array}}^{n}{\left(c_{i}^{q}+c_{j}^{q}-c_{i}^{q}c_{j}^{q}\right)}^{\frac{1}{n(n+1)}}\right)}^{\frac{1}{2}}\right)}^{1/q},{\left(1-{\left(1-\prod_{\begin{array}{l} i,j=1\\ i\neq j \end{array}}^{n}{\left(d_{i}^{q}+d_{j}^{q}-d_{i}^{q}d_{j}^{q}\right)}^{\frac{1}{n(n+1)}}\right)}^{\frac{1}{2}}\right)}^{1/q}])\rangle .$$

$$={\left(\frac{1}{n(n-1)}\overset{n}{\underset{\begin{array}{l} i,j=1\\ i\neq j \end{array}}{⊕}}(\alpha_{i}⊗\alpha_{j})\right)}^{\frac{1}{2}}.$$ (39)

**Special case 3:** if $H=\overset{k}{\overset{︷}{(1,1,\ldots ,1,}}\overset{n-k}{\overset{︷}{0,0,\ldots ,0)}}$, then the IVq-ROULPMM operator reduces to the interval-valued q-rung orthopair uncertain linguistic power Maclaurin symmetric mean (IVq-ROULPMSM) operator, i.e.,

$$IVq-ROULPMM^{\overset{k}{\overset{︷}{\left(1,1,\ldots ,1\right)}}\overset{n-k}{\overset{︷}{),0,0,\ldots ,0)}}}(\alpha_{1},\alpha_{2},\ldots ,\alpha_{n})=$$

$$\langle [f{*}^{-1}({\left(1-\prod_{1\leq i_{1}<i_{2}<\ldots <i_{k}\leq n}{\left(1-\prod_{j=1}^{k}\left(1-{\left(1-f{*}^{-1}(\theta_{i_{j}})\right)}^{n\varpi_{i_{j}}}\right)\right)}^{\frac{1}{C_{n}^{k}}}\right)}^{\frac{1}{k}}),$$

$$f{*}^{-1}({\left(1-\prod_{1\leq i_{1}<i_{2}<\ldots <i_{k}\leq n}{\left(1-\prod_{j=1}^{k}\left(1-{\left(1-f{*}^{-1}(\tau_{i_{j}})\right)}^{n\varpi_{i_{j}}}\right)\right)}^{\frac{1}{C_{n}^{k}}}\right)}^{\frac{1}{k}})],$$

$$([{\left(1-\prod_{1\leq i_{1}<i_{2}<\ldots <i_{k}\leq n}{\left(1-\prod_{j=1}^{k}\left(1-{\left(1-a_{i_{j}}^{q}\right)}^{n\varpi_{i_{j}}}\right)\right)}^{\frac{1}{C_{n}^{k}}}\right)}^{\frac{1}{qk}},{\left(1-\prod_{1\leq i_{1}<i_{2}<\ldots <i_{k}\leq n}{\left(1-\prod_{j=1}^{k}\left(1-{\left(1-b_{i_{j}}^{q}\right)}^{n\varpi_{i_{j}}}\right)\right)}^{\frac{1}{C_{n}^{k}}}\right)}^{\frac{1}{qk}}],$$

$$\left[{\left(1-{\left(1-\prod_{1\leq i_{1}<i_{2}<\ldots <i_{k}\leq n}{\left(1-\prod_{j=1}^{k}\left(1-{\left(c_{i_{j}}^{n\varpi_{i_{j}}}\right)}^{q}\right)\right)}^{\frac{1}{C_{n}^{k}}}\right)}^{\frac{1}{k}}\right)}^{1/q},{\left(1-{\left(1-\prod_{1\leq i_{1}<i_{2}<\ldots <i_{k}\leq n}{\left(1-\prod_{j=1}^{k}\left(1-{\left(d_{i_{j}}^{n\varpi_{i_{j}}}\right)}^{q}\right)\right)}^{\frac{1}{C_{n}^{k}}}\right)}^{\frac{1}{k}}\right)}^{1/q}])\right\rangle$$

$$={\left(\frac{1}{C_{n}^{k}}\underset{1\leq i_{1}<i_{2}<\ldots <i_{k}\leq n}{⊕}\overset{k}{\underset{j=1}{⊗}}(n\varpi_{i_{j}}\alpha_{i_{j}})\right)}^{\frac{1}{k}}.$$ (40)

In this case, if *Sup*(*α*_*i*_,*α*_*j*_) = *t*>0 for all *i*≠*j*, then the IVq-ROULPMM operator reduces to the interval-valued q-rung orthopair uncertain linguistic Maclaurin symmetric mean (IVq-ROULMSM) operator, i.e.,

$$IVq-ROULPMM^{\overset{k}{\overset{︷}{(1,1,\ldots ,1}}\overset{n-k}{\overset{︷}{,0,0,\ldots ,0)}}}(\alpha_{1},\alpha_{2},\ldots ,\alpha_{n})=$$

$$\langle [f{*}^{-1}({\left(1-\prod_{1\leq i_{1}<i_{2}<\ldots <i_{k}\leq n}{\left(1-\prod_{j=1}^{k}f*(\theta_{i_{j}})\right)}^{\frac{1}{C_{n}^{k}}}\right)}^{\frac{1}{k}}),f{*}^{-1}({\left(1-\prod_{1\leq i_{1}<i_{2}<\ldots <i_{k}\leq n}{\left(1-\prod_{j=1}^{k}f*(\tau_{i_{j}})\right)}^{\frac{1}{C_{n}^{k}}}\right)}^{\frac{1}{k}})],$$

$$([{\left(1-\prod_{1\leq i_{1}<i_{2}<\ldots <i_{k}\leq n}{\left(1-\prod_{j=1}^{k}a_{i_{j}}^{q}\right)}^{\frac{1}{C_{n}^{k}}}\right)}^{\frac{1}{qk}},{\left(1-\prod_{1\leq i_{1}<i_{2}<\ldots <i_{k}\leq n}{\left(1-\prod_{j=1}^{k}b_{i_{j}}^{q}\right)}^{\frac{1}{C_{n}^{k}}}\right)}^{\frac{1}{qk}}],$$

$$\left[{\left(1-{\left(1-\prod_{1\leq i_{1}<i_{2}<\ldots <i_{k}\leq n}{\left(1-\prod_{j=1}^{k}\left(1-c_{i_{j}}^{q}\right)\right)}^{\frac{1}{C_{n}^{k}}}\right)}^{\frac{1}{k}}\right)}^{1/q},{\left(1-{\left(1-\prod_{1\leq i_{1}<i_{2}<\ldots <i_{k}\leq n}{\left(1-\prod_{j=1}^{k}\left(1-d_{i_{j}}^{q}\right)\right)}^{\frac{1}{C_{n}^{k}}}\right)}^{\frac{1}{k}}\right)}^{1/q}])\right\rangle$$

$$={\left(\frac{1}{C_{n}^{k}}\underset{1\leq i_{1}<i_{2}<\ldots <i_{k}\leq n}{⊕}\overset{k}{\underset{j=1}{⊗}}(\alpha_{i_{j}})\right)}^{\frac{1}{k}}.$$ (41)

**Special case 4:** if *H* = (1,1,….1) or (1/*n*,1/*n*,…,1/*n*), then the IVq-ROULPMM operator reduces to the following form

$$IVq-ROULPMM^{\left(1,1,\ldots ,1)\;\mathrm{or}\;(1/n,1/n,\ldots ,1/n\right)}(\alpha_{1},\alpha_{2},\ldots ,\alpha_{n})=$$

$$\langle [f{*}^{-1}(\prod_{j=1}^{n}{\left(1-{\left(1-f*(\theta_{j})\right)}^{n\varpi_{j}}\right)}^{\frac{1}{n}}),f{*}^{-1}(f{*}^{-1}(\prod_{j=1}^{n}{\left(1-{\left(1-f*(\tau_{j})\right)}^{n\varpi_{j}}\right)}^{\frac{1}{n}}))],$$

$$([({\overset{n}{\underset{j=1}{\prod }}(1-{\left(1-a_{j}^{q}\right)}^{n\sigma_{j}}))}^{\frac{1}{nq}},{\left(\overset{n}{\underset{j=1}{\prod }}(1-{\left(1-b_{j}^{q}\right)}^{n\sigma_{j}})\right)}^{\frac{1}{nq}}],$$

$$\left[{\left(1-\prod_{j=1}^{n}{\left(1-{\left(c_{j}^{n\varpi_{j}}\right)}^{q}\right)}^{\frac{1}{n}}\right)}^{1/q},{\left(1-\prod_{j=1}^{n}{\left(1-{\left(d_{j}^{n\varpi_{j}}\right)}^{q}\right)}^{\frac{1}{n}}\right)}^{1/q}\right])\rangle =\overset{n}{\underset{j=1}{⊗}}{\left(n\varpi_{j}\alpha_{j}\right)}^{\frac{1}{n}}.$$ (42)

In this case, if *Sup*(*α*_*i*_,*α*_*j*_) = *t*>0 for all *i*≠*j*, then the IVq-ROULPMM operator reduces to the interval-valued q-rung orthopair uncertain linguistic geometric (IVq-ROULG) operator, i.e.,

$$IVq-ROULPMM^{\left(1,1,\ldots ,1)\;\mathrm{or}\;(1/n,1/n,\ldots ,1/n\right)}(\alpha_{1},\alpha_{2},\ldots ,\alpha_{n})=\langle [f{*}^{-1}(\prod_{j=1}^{n}{\left(f*(\theta_{j})\right)}^{\frac{1}{n}}),f{*}^{-1}(\prod_{j=1}^{n}{\left(f*(\tau_{j})\right)}^{\frac{1}{n}})],$$

$$(\left[{\left(\prod_{j=1}^{n}\left(a_{j}\right)\right)}^{\frac{1}{n}},{\left(\prod_{j=1}^{n}\left(b_{j}\right)\right)}^{\frac{1}{n}}],[{\left(1-\prod_{j=1}^{n}{\left(1-c_{j}^{q}\right)}^{\frac{1}{n}}\right)}^{\frac{1}{q}},{\left(1-\prod_{j=1}^{n}{\left(1-d_{j}^{q}\right)}^{\frac{1}{n}}\right)}^{\frac{1}{q}}\right])=\overset{n}{\underset{j=1}{⊗}}\alpha_{j}^{1/n}.$$ (43)

**Special case 5:** if *q* = 2, then the IVq-ROULPMM operator reduces to the interval-valued Pythagorean uncertain linguistic power Muirhead mean (IVPULPMM) operator, i.e.,

$$IVq-ROULPMM^{H}(\alpha_{1},\alpha_{2},\ldots ,\alpha_{n})=\langle [f{*}^{-1}({\left(1-\prod_{\varsigma \in T_{n}}{\left(1-\prod_{j=1}^{n}{\left(1-{\left(1-f*(\theta_{\varsigma (j)})\right)}^{n\varpi_{\varsigma (j)}}\right)}^{h_{j}}\right)}^{\frac{1}{n!}}\right)}^{\frac{1}{\sum_{j=1}^{n}h_{j}}}),$$

$$f{*}^{-1}({\left(1-\prod_{\varsigma \in T_{n}}{\left(1-\prod_{j=1}^{n}{\left(1-{\left(1-f*(\tau_{\varsigma (j)})\right)}^{n\varpi_{\varsigma (j)}}\right)}^{h_{j}}\right)}^{\frac{1}{n!}}\right)}^{\frac{1}{\sum_{j=1}^{n}h_{j}}})],$$

$$([{\left(1-\prod_{\varsigma \in T_{n}}{\left(1-\prod_{j=1}^{n}{\left(1-{\left(1-a_{\varsigma (j)}^{2}\right)}^{n\varpi_{\varsigma (j)}}\right)}^{h_{j}}\right)}^{\frac{1}{n!}}\right)}^{\frac{1}{2\sum_{j=1}^{n}h_{j}}},{\left(1-\prod_{\varsigma \in T_{n}}{\left(1-\prod_{j=1}^{n}{\left(1-{\left(1-b_{\varsigma (j)}^{2}\right)}^{n\varpi_{\varsigma (j)}}\right)}^{h_{j}}\right)}^{\frac{1}{n!}}\right)}^{\frac{1}{2\sum_{j=1}^{n}h_{j}}}],$$

$$\left[{\left(1-{\left(1-\prod_{\varsigma \in T_{n}}{\left(1-\prod_{j=1}^{n}{\left(1-{\left(c_{\varsigma (j)}^{n\varpi_{\varsigma (j)}}\right)}^{2}\right)}^{h_{j}}\right)}^{\frac{1}{n!}}\right)}^{\frac{1}{\sum_{j=1}^{n}h_{j}}}\right)}^{1/2},{\left(1-{\left(1-\prod_{\varsigma \in T_{n}}{\left(1-\prod_{j=1}^{n}{\left(1-{\left(d_{\varsigma (j)}^{n\varpi_{\varsigma (j)}}\right)}^{2}\right)}^{h_{j}}\right)}^{\frac{1}{n!}}\right)}^{\frac{1}{\sum_{j=1}^{n}h_{j}}}\right)}^{1/2}])\right\rangle$$

$$=IVPULPMM^{H}(\alpha_{1},\alpha_{2},\ldots ,\alpha_{n}).$$ (44)

**Special case 6:** if *q* = 1, then the IVq-ROULPMM operator reduces to the interval-valued intuitionistic uncertain linguistic power Muirhead mean (IVIULPMM) operator, i.e.,

$$IVq-ROULPMM^{H}(\alpha_{1},\alpha_{2},\ldots ,\alpha_{n})=\langle [f{*}^{-1}({\left(1-\prod_{\varsigma \in T_{n}}{\left(1-\prod_{j=1}^{n}{\left(1-{\left(1-f*(\theta_{\varsigma (j)})\right)}^{n\varpi_{\varsigma (j)}}\right)}^{h_{j}}\right)}^{\frac{1}{n!}}\right)}^{\frac{1}{\sum_{j=1}^{n}h_{j}}}),$$

$$f{*}^{-1}({\left(1-\prod_{\varsigma \in T_{n}}{\left(1-\prod_{j=1}^{n}{\left(1-{\left(1-f*(\tau_{\varsigma (j)})\right)}^{n\varpi_{\varsigma (j)}}\right)}^{h_{j}}\right)}^{\frac{1}{n!}}\right)}^{\frac{1}{\sum_{j=1}^{n}h_{j}}})],$$

$$(\left[{\left(1-\prod_{\varsigma \in T_{n}}{\left(1-\prod_{j=1}^{n}{\left(1-{\left(1-a_{\varsigma (j)}^{}\right)}^{n\varpi_{\varsigma (j)}}\right)}^{h_{j}}\right)}^{\frac{1}{n!}}\right)}^{\frac{1}{\sum_{j=1}^{n}h_{j}}},{\left(1-\prod_{\varsigma \in T_{n}}{\left(1-\prod_{j=1}^{n}{\left(1-{\left(1-b_{\varsigma (j)}^{}\right)}^{n\varpi_{\varsigma (j)}}\right)}^{h_{j}}\right)}^{\frac{1}{n!}}\right)}^{\frac{1}{\sum_{j=1}^{n}h_{j}}}\right],$$

$$\left[\left(1-{\left(1-\prod_{\varsigma \in T_{n}}{\left(1-\prod_{j=1}^{n}{\left(1-c_{\varsigma (j)}^{n\varpi_{\varsigma (j)}}\right)}^{h_{j}}\right)}^{\frac{1}{n!}}\right)}^{\frac{1}{\sum_{j=1}^{n}h_{j}}}),(1-{\left(1-\prod_{\varsigma \in T_{n}}{\left(1-\prod_{j=1}^{n}{\left(1-d_{\varsigma (j)}^{n\varpi_{\varsigma (j)}}\right)}^{h_{j}}\right)}^{\frac{1}{n!}}\right)}^{\frac{1}{\sum_{j=1}^{n}h_{j}}}\right)])\right\rangle$$

$$=IVIULPMM^{H}(\alpha_{1},\alpha_{2},\ldots ,\alpha_{n}).$$ (45)

### 5.4 The interval-valued q-rung orthopair uncertain linguistic power weighted Muirhead mean operator

**Definition 15**. Let *α*_*j*_(*j* = 1,2,…,*n*) be a series of IVq-ROULVs, and *H* = (*h*_1_,*h*_2_,…,*h*_*n*_)∈*R*^*n*^ be a set of parameters. Let *w* = (*w*_1_,*w*_2_,…,*w*_*n*_)^*T*^ be the weight vector, such that 0≤*w*_*j*_≤1 and $\sum_{j=1}^{n}w_{j}=1$. The interval-valued q-rung orthopair uncertain linguistic power weighted Muirhead mean (IVq-ROULPWMM) operator is defined as

$$IVq-ROULPWMM^{H}(\alpha_{1},\alpha_{2},\ldots ,\alpha_{n})={\left(\frac{1}{n!}\underset{\zeta \in T_{n}}{⊕}\overset{n}{\underset{j=1}{⊗}}{\left(n\frac{w_{\zeta (j)}(1+T(\alpha_{\zeta (j)}))}{\sum_{j=1}^{n}w_{j}(1+T(\alpha_{j}))}\alpha_{\zeta (j)}\right)}^{h_{j}}\right)}^{\frac{1}{\sum_{j=1}^{n}h_{j}}},$$ (46)

where

$$T(\alpha_{j})=\sum_{j=1.j\neq i}^{n}Sup(\alpha_{i},\alpha_{j}),$$ (47)

and

$$Sup(\alpha_{i},\alpha_{j})=1-d(\alpha_{i},\alpha_{j}),$$ (48)

where *ζ*(*j*)(*j* = 1,2,…,*n*) denotes any permutation of (1,2,…,*n*), *T*_*n*_ represents all possible permutations of (1,2,…,*n*), and *n* is the balancing coefficient. *d*(*α*_*i*_,*α*_*j*_) represents the Hamming distance between *α*_*i*_ and *α*_*j*_, and *Sup*(*α*_*i*_,*α*_*j*_) is the support for *α*_*i*_ from *α*_*j*_, satisfying the properties presented in Definition 12.

To simiplify Eq (46), let

$$\delta_{j}=\frac{w_{j}(1+T(\alpha_{j}))}{\sum_{j=1}^{n}w_{j}(1+T(\alpha_{j}))},$$ (49)

then, Eq (46) can be written as

$$IVq-ROULPWMM^{H}(\alpha_{1},\alpha_{2},\ldots ,\alpha_{n})={\left(\frac{1}{n!}\underset{\varsigma \in T_{n}}{⊕}\overset{n}{\underset{j=1}{⊗}}{\left(n\delta_{\varsigma (j)}\alpha_{\varsigma (j)}\right)}^{h_{j}}\right)}^{\frac{1}{\sum_{j=1}^{n}h_{j}}},$$ (50)

where 0≤*δ*_*j*_≤1 and $\sum_{j=1}^{n}\delta_{j}=1$.

**Theorem 9.** Let $\alpha_{j}=\langle \left[s_{\theta_{j}},s_{\tau_{j}}],(\left[a_{j},b_{j}],[c_{j},d_{j}\right]\right)\rangle$ (j=1,2,…,n) be a series of IVq-ROULVs, then the aggregated value by the IVq-ROULPWMM operator is still an IVq-ROULV and

$$IVq-ROULPWMM^{H}(\alpha_{1},\alpha_{2},\ldots ,\alpha_{n})=\langle [f{*}^{-1}({\left(1-\prod_{\varsigma \in T_{n}}{\left(1-\prod_{j=1}^{n}{\left(1-{\left(1-f*(\theta_{\varsigma (j)})\right)}^{n\delta_{\varsigma (j)}}\right)}^{h_{j}}\right)}^{\frac{1}{n!}}\right)}^{\frac{1}{\sum_{j=1}^{n}h_{j}}}),$$

$$f{*}^{-1}({\left(1-\prod_{\varsigma \in T_{n}}{\left(1-\prod_{j=1}^{n}{\left(1-{\left(1-f*(\tau_{\varsigma (j)})\right)}^{n\delta_{\varsigma (j)}}\right)}^{h_{j}}\right)}^{\frac{1}{n!}}\right)}^{\frac{1}{\sum_{j=1}^{n}h_{j}}})],$$

$$([{\left(1-\prod_{\varsigma \in T_{n}}{\left(1-\prod_{j=1}^{n}{\left(1-{\left(1-a_{\varsigma (j)}^{q}\right)}^{n\delta_{\varsigma (j)}}\right)}^{h_{j}}\right)}^{\frac{1}{n!}}\right)}^{\frac{1}{q\sum_{j=1}^{n}h_{j}}},{\left(1-\prod_{\varsigma \in T_{n}}{\left(1-\prod_{j=1}^{n}{\left(1-{\left(1-b_{\varsigma (j)}^{q}\right)}^{n\delta_{\varsigma (j)}}\right)}^{h_{j}}\right)}^{\frac{1}{n!}}\right)}^{\frac{1}{q\sum_{j=1}^{n}h_{j}}}],$$

$$[{\left(1-{\left(1-\prod_{\varsigma \in T_{n}}{\left(1-\prod_{j=1}^{n}{\left(1-{\left(c_{\varsigma (j)}^{n\delta_{\varsigma (j)}}\right)}^{q}\right)}^{h_{j}}\right)}^{\frac{1}{n!}}\right)}^{\frac{1}{\sum_{j=1}^{n}h_{j}}}\right)}^{1/q},{\left(1-{\left(1-\prod_{\varsigma \in T_{n}}{\left(1-\prod_{j=1}^{n}{\left(1-{\left(d_{\varsigma (j)}^{n\delta_{\varsigma (j)}}\right)}^{q}\right)}^{h_{j}}\right)}^{\frac{1}{n!}}\right)}^{\frac{1}{\sum_{j=1}^{n}h_{j}}}\right)}^{1/q}])\rangle ,$$ (51)

The proof of Theorem 9 is similar to that of Theorem 6. In addition, it is easy to prove that the IVq-ROULPWMM operator has the property of boundedness.

## 6 A novel method to MAGDM with interval-valued q-rung orthopair uncertain linguistic information

In the above sections, we have built the information representation and fusion method of MAGDM problems. In this section, we construct a complete model to show the process of applying the proposed method to real MAGDM problems. Consider a MAGDM problem evaluated by IVq-ROUL information. Suppose there are *m* alternatives *A* = {*A*_1_,*A*_2_,…,*A*_*m*_} that to be evaluated. Let *C* = {*C*_1_,*C*_2_,…,*C*_*n*_} be a set of attributes, whose weight vector is *w* = (*w*_1_,*w*_2_,…,*w*_*n*_)^*T*^, satisfying the condition that $\sum_{l=1}^{n}w_{l}=1$ and 0≤*w*_*i*_≤1. Let *D* =(*D*_1_,*D*_2_,…,*D*_*t*_} be a set of DMs with the weight vector being *γ* = (*γ*_1_,*γ*_2_,…,*γ*_*t*_)^*T*^, such that 0≤*γ*_*k*_≤1 and $\sum_{k=1}^{t}\gamma_{k}=1$. The DM *D*_*s*_ employs an IVq-ROULV $\alpha_{ij}^{s}=\langle \left[s_{\theta_{ij}^{s}},s_{\tau_{ij}^{s}}],(\left[a_{ij}^{s},b_{ij}^{s}],[c_{ij}^{s},d_{ij}^{s}\right]\right)\rangle$ to express his/her evaluation value with regard to alternative *A*_*i*_(*i* = 1,2,…,*m*) under attribute *C*_*j*_(*j* = 1,2,…,*n*). Hence, a set of IVq-ROUL decision matrices $R^{s}={\left(\alpha_{ij}^{s}\right)}_{m\times n}$ are obtained. In the followings, we provide a complete process of selecting the best alternative according to the proposed method.

**Step 1:** Before taking calculations, we should first normalize the original decision matrices according to the characteristics of the attributes, and convert all of them to the benefit type according to the following formula

$$\alpha_{ij}^{s}=\{\begin{array}{l} \langle \left[s_{\theta_{ij}^{s}},s_{\tau_{ij}^{s}}],(\left[a_{ij}^{s},b_{ij}^{s}],[c_{ij}^{s},d_{ij}^{s}\right]\right)\rangle ,G_{j}\;is\;benefit\;type\\ \langle \left[s_{\theta_{ij}^{s}},s_{\tau_{ij}^{s}}],(\left[c_{ij}^{s},d_{ij}^{s}],[a_{ij}^{s},b_{ij}^{s}\right]\right)\rangle ,G_{j}\;is\;cost\;type \end{array},$$ (52)

**Step 2:** Calculate the supports $Sup(\alpha_{ij}^{k},\alpha_{ij}^{d})$ according to the following

$$Sup(\alpha_{ij}^{k},\alpha_{ij}^{d})=1-d(\alpha_{ij}^{k},\alpha_{ij}^{d}),$$ (53)

where *k*,*d* = 1,2,…,*t*;*k*≠*d*;*i* = 1,2…,*m*;*j* = 1,2,…,*n* and $d(\alpha_{ij}^{k},\alpha_{ij}^{d})$ is the Hamming distance between $\alpha_{ij}^{k}$ and $\alpha_{ij}^{d}$.

**Step 3:** Calculate $T(\alpha_{ij}^{k})$ by

$$T(\alpha_{ij}^{k})=\sum_{k=1,k\neq d}^{n}Sup(\alpha_{ij}^{k},\alpha_{ij}^{d}),$$ (54)

where *k*,*d* = 1,2,…,*t*;*i* = 1,2…,*m*;*j* = 1,2,…,*n*.

**Step 4:** Compute the power weight $\delta_{ij}^{k}$ associated with the IVq-ROULV $\alpha_{ij}^{k}$ by

$$\delta_{ij}^{k}=\frac{\gamma_{k}(1+T(\alpha_{ij}^{k}))}{\sum_{k=1}^{t}\gamma_{k}(1+T(\alpha_{ij}^{k}))},$$ (55)

where $k=1,2,\ldots ,t;i=1,2\ldots ,m;j=1,2,\ldots ,n,\delta_{ij}^{k}>0$ and $\sum_{k=1}^{t}\delta_{ij}^{k}=1$.

**Step 5:** Utilized the IVq-ROULPWA operator to aggregate individual decision matrix, i.e.,

$$\alpha_{ij}=IVq-ROULPWA(\alpha_{ij}^{1},\alpha_{ij}^{2},\ldots ,\alpha_{ij}^{t}),$$ (56)

**Step 6:** Calculate the supports $Sup(\alpha_{il}^{k},\alpha_{if}^{d})$ by

$$Sup(\alpha_{il}^{k},\alpha_{if}^{d})=1-d(\alpha_{il}^{k},\alpha_{if}^{d}),$$ (57)

where *i* = 1,2,…,*n*;*l*,*f* = 1,2,…,*n*;*l*≠*f* and *d*(*α*_*il*_,*α*_*if*_) is the Hamming distance between *α*_*il*_ and *α*_*if*_.

**Step 7:** Compute *T*(*α*_*ij*_) by

$$T(\alpha_{ij}^{k})=\sum_{f,l=1,f=1}^{n}Sup(\alpha_{il},\alpha_{if}),$$ (58)

where *i* = 1,2,…,*m*;*l*,*f* = 1,2,…,*n*.

**Step 8:** Calculate the power weight *η*_*ij*_ associated with IVq-ROULV *α*_*ij*_ according to the following

$$\eta_{ij}=\frac{w_{j}(1+T(\alpha_{ij}))}{\sum_{j=1}^{n}w_{j}(1+T(\alpha_{ij}))},$$ (59)

where *i* = 1,2,…,*m*;*j* = 1,2,…,*n*.

**Step 9:** For alternative *A*_*i*_(*i* = 1,2,…,*n*), utilize the IVq-ROULPWMM operator

$$\alpha_{ij}=IVq-ROULPWMM^{H}(\alpha_{i1},\alpha_{i2},\ldots ,\alpha_{in}),$$ (60)

to aggregate attributes, and get the overall evaluation value.

**Step 10:** Compute scores of overall evaluations.

**Step 11:** Rank the alternatives and obtain the final result.

**Fig 1:**
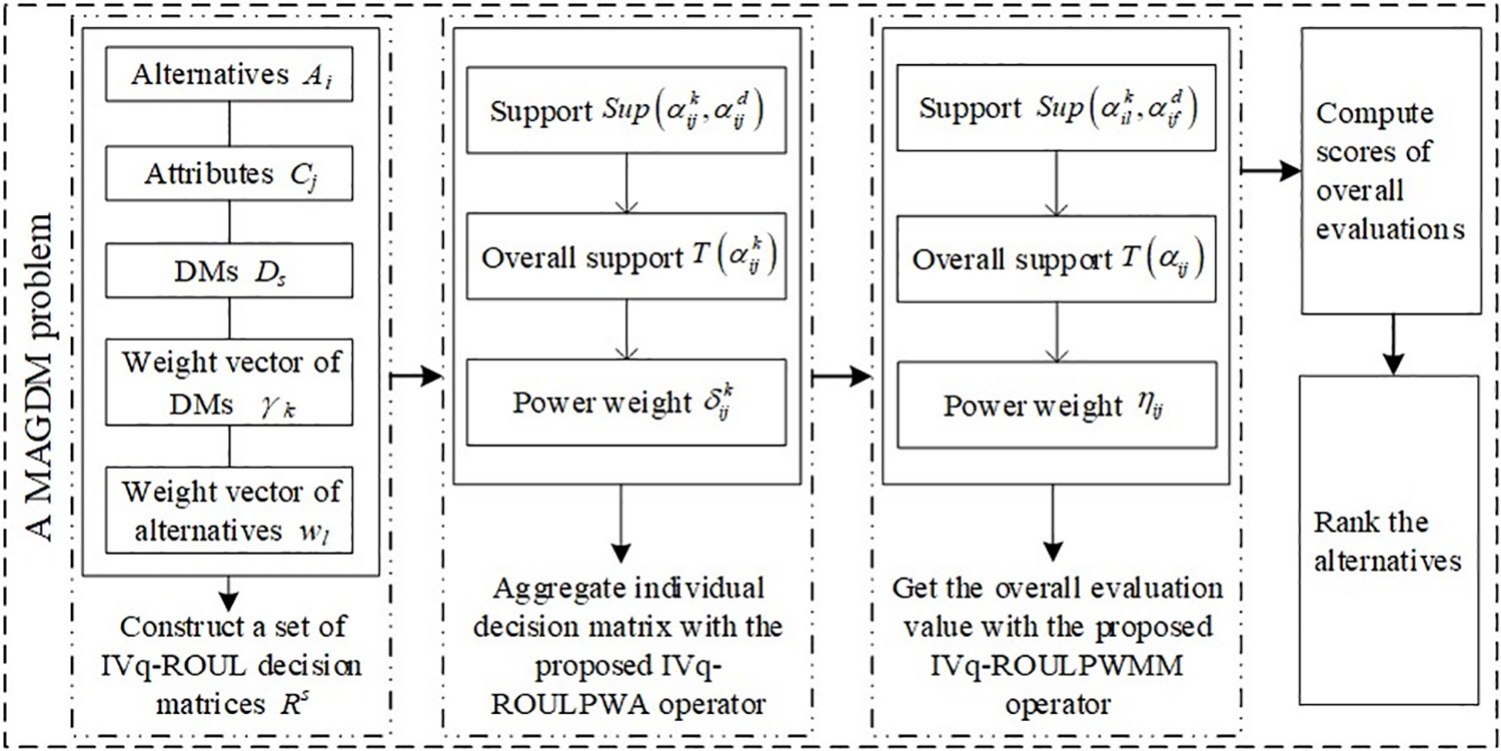
The flowchart of our proposed MAGDM method.

To better illustrate the decision-making process with the proposed method, we give the flowchart in Fig 1.

## 7 An application of the proposed method in downward referral hospital evaluation

China’s new medical reform strategy clearly proposed to establish the system of first treatment in the community, hospital two-way referral and hierarchical diagnosis and treatment. More than a decade of practices show that it is easy to upward-referral but hard to downward-referral in the implementation process of the two-way referral. Thus, more and more scholars have been appealed to explore how to improve the downward referral rate of hospital in recent years. For patients, different conditions mean different demands for medical equipment and medical conditions requirements. Therefore, it is necessary for physicians to conduct a detailed assessment of the hospital to which the patients are eventually transferred. Affected by many factors, the selection of downward referral hospital has a high degree of uncertainty. To this end, this paper provides the following example to illustrate the process of how the proposed method can be utilized to solve the problem of the selection of hospitals in the downward referral.

**Example 5.** Assume that one patient needs to be referred to a subordinate hospital and there are currently four alternative hospitals *A*_*i*_(*i* =1,2,3,4), three physicians *D*_*t*_(*t* = 1,2,3) are invited to evaluate the hospitals with respect to four indicators: (1) doctor’s level of medical satisfaction *C*_1_; (2) medical facility satisfaction *C*_2_ (3) hospital drug supply satisfaction *C*_3_; (4) the degree of cooperation with the hospital *C*_4_. The weighted vector of attributes is *w* = (0.27,0.19,0.21,0.33)^*T*^. Three physicians *D*_*t*_(*t* = 1,2,3) are invited to evaluate the four attributes *C*_*j*_(*j* = 1,2,3,4) with interval-valued *q*-rung orthopair uncertain linguistic information. Weight vector of DMs is *γ* = (0.30,0.40,0.30)^*T*^, and the decision matrices are shown in Tables 2–4. With the evaluations given by DMs, we assess the scores of all hospitals’ conditions with the proposed method, and the higher the score, the most likely to be chosen. It should be noted that physicians can assess whether the hospital meets the requirements to be selected or not by linguistic set *S* ={*s*_1_,*s*_2_,*s*_3_,*s*_4_,*s*_5_,*s*_6_}, and DMs’ attitude of approval gradually gets stronger from *s*_1_ to *s*_6_.

**Table 2:** The interval-valued q-rung orthopair uncertain linguistic decision matrix *R*^1^ given by *D*_1_.

	*C*_1_	*C*_2_	*C*_3_	*C*_4_
*A*_1_	<[*s*_3_, *s*_5_], ([0.6, 0.8], [0.1, 0.2])>	<[*s*_2_, *s*_3_], ([0.4, 0.5], [0.2, 0.3])>	<[*s*_4_, *s*_6_], ([0.6, 0.7], [0.1, 0.1])>	<[*s*_3_, *s*_5_], ([0.5, 0.8], [0.1, 0.2])>
A_2_	<[*s*_3_, *s*_5_], ([0.5, 0.6], [0.1, 0.2])>	<[*s*_3_, *s*_6_], ([0.4, 0.7], [0.2, 0.2])>	<[*s*_4_, *s*_6_], ([0.3, 0.4], [0.1, 0.3])>	<[*s*_3_, *s*_5_], ([0.5, 0.7], [0.2, 0.3])>
A_3_	<[*s*_3_, *s*_4_], ([0.5, 0.7], [0.1, 0.2])>	<[*s*_2_, *s*_3_], ([0.4, 0.5], [0.3, 0.4])>	<[*s*_3_, *s*_5_], ([0.4, 0.7], [0.1, 0.2])>	<[*s*_5_, *s*_6_], ([0.7, 0.8], [0.1, 0.2])>
A_4_	<[*s*_3_, *s*_4_], ([0.6, 0.7], [0.2, 0.3])>	<[*s*_1_, *s*_3_], ([0.5, 0.6], [0.3, 0.4])>	<[*s*_4_, *s*_6_], ([0.3, 0.4], [0.2, 0.3])>	<[*s*_3_, *s*_4_], ([0.5, 0.7], [0.1, 0.2])>

**Table 3:** The interval-valued q-rung orthopair uncertain linguistic decision matrix *R*^2^ given by *D*_2_.

	*C*_1_	*C*_2_	*C*_3_	*C*_4_
*A*_1_	<[*s*_3_, *s*_5_], ([0.4, 0.7], [0.1, 0.2])>	<[*s*_4_, *s*_6_], ([0.6, 0.7], [0.2, 0.3])>	<[*s*_3_, *s*_4_], ([0.3, 0.5], [0.3, 0.4])>	<[*s*_4_, *s*_5_], ([0.6, 0.8], [0.1, 0.2])>
A_2_	<[*s*_3_, *s*_5_], ([0.4, 0.5], [0.3, 0.4])>	<[*s*_5_, *s*_6_], ([0.4, 0.8], [0.1, 0.2])>	<[*s*_4_, *s*_5_], ([0.6, 0.7], [0.2, 0.3])>	<[*s*_3_, *s*_4_], ([0.5, 0.6], [0.1, 0.2])>
A_3_	<[*s*_4_, *s*_6_], ([0.3, 0.4], [0.1, 0.3])>	<[*s*_3_, *s*_5_], ([0.4, 0.7], [0.1, 0.2])>	<[*s*_5_, *s*_6_], ([0.5, 0.7], [0.1, 0.2])>	<[*s*_3_, *s*_5_], ([0.5, 0.6], [0.2, 0.3])>
A_4_	<[*s*_3_, *s*_4_], ([0.5, 0.6], [0.2, 0.3])>	<[*s*_3_, *s*_4_], ([0.4, 0.5], [0.3, 0.4])>	<[*s*_3_, *s*_4_], ([0.5, 0.7], [0.1, 0.2])>	<[*s*_3_, *s*_4_], ([0.5, 0.6], [0.2, 0.3])>

**Table 4:** The interval-valued q-rung orthopair uncertain linguistic decision matrix *R*^3^ given by *D*_3_.

	*C*_1_	*C*_2_	*C*_3_	*C*_4_
*A*_1_	<[*s*_2_, *s*_3_], ([0.5, 0.6], [0.2, 0.3])>	<[*s*_4_, *s*_5_], ([0.7, 0.8], [0.1, 0.2])>	<[*s*_5_, *s*_6_], ([0.5, 0.7], [0.1, 0.3])>	<[*s*_3_, *s*_4_], ([0.5, 0.7], [0.1, 0.2])>
A_2_	<[*s*_3_, *s*_4_], ([0.5, 0.6], [0.2, 0.2])>	<[*s*_2_, *s*_5_], ([0.4, 0.7], [0.1, 0.2])>	<[*s*_3_, *s*_4_], ([0.5, 0.7], [0.1, 0.2])>	<[*s*_3_, *s*_6_], ([0.4, 0.7], [0.1, 0.2])>
A_3_	<[*s*_2_, *s*_3_], ([0.3, 0.4], [0.4, 0.5])>	<[*s*_2_, *s*_5_], ([0.5, 0.7], [0.1, 0.2])>	<[*s*_3_, *s*_4_], ([0.5, 0.6], [0.2, 0.3])>	<[*s*_5_, *s*_6_], ([0.4, 0.6], [0.1, 0.2])>
A_4_	<[*s*_5_, *s*_6_], ([0.4, 0.6], [0.2, 0.3])>	<[*s*_3_, *s*_4_], ([0.5, 0.6], [0.2, 0.3])>	<[*s*_5_, *s*_6_], ([0.6, 0.8], [0.1, 0.2])>	<[*s*_4_, *s*_5_], ([0.7, 0.8], [0.1, 0.2])>

### 7.1 The decision-making process

**Step 1:** It is clearly that all attributes are benefit type, there is no need to normalize the original decision matrix.

**Step 2:** Calculate the $Sup(\alpha_{ij}^{k},\alpha_{ij}^{d})$ according to Eq (53) (Suppose that *q* = 3 and LFS1 is utilized as the specified LSF in the calculation process). For convenience, the support between $\alpha_{ij}^{k}$ and $\alpha_{ij}^{d}$ are expressed in $S_{d}^{k}$, where *i*,*j* = 1,2,3,4;*k*,*d* = 1,2,3;*k*≠*d*. Then, we can obtain

$$S_{2}^{1}=S_{1}^{2}=[\begin{array}{cccc} 0.9331 & 0.9152 & 0.8863 & 0.9791\\ 0.9513 & 0.9487 & 0.8911 & 0.9713\\ 0.9093 & 0.9438 & 0.9835 & 0.8538\\ 0.9637 & 0.9715 & 0.9076 & 0.9745 \end{array}]$$

$$S_{3}^{1}=S_{1}^{3}=[\begin{array}{cccc} 0.9226 & 0.8558 & 0.9683 & 0.9683\\ 0.9987 & 0.9983 & 0.9093 & 0.9801\\ 0.9072 & 0.9398 & 0.9599 & 0.8563\\ 0.9303 & 0.9895 & 0.8204 & 0.9194 \end{array}]$$

$$S_{3}^{2}=S_{2}^{3}=[\begin{array}{cccc} 0.9599 & 0.9262 & 0.9053 & 0.9458\\ 0.9574 & 0.9507 & 0.9756 & 0.9608\\ 0.9631 & 0.9860 & 0.9618 & 0.9764\\ 0.9848 & 0.9653 & 0.9350 & 0.8875 \end{array}]$$

**Step 3:** Calculate $T(\alpha_{ij}^{k})$ according to Eq (54). For convenience, $T(\alpha_{ij}^{k})$ are expressed in *T*^*k*^, where *i*,*j* = 1,2,3,4;*k* = 1,2,3. Then, we can obtain

$$T^{1}=[\begin{array}{cccc} 1.8557 & 1.7710 & 1.8546 & 1.9475\\ 1.9499 & 1.9469 & 1.8004 & 1.9514\\ 1.8164 & 1.8836 & 1.9434 & 1.7100\\ 1.8939 & 1.9610 & 1.7281 & 1.8939 \end{array}].$$

$$T^{2}=[\begin{array}{cccc} 1.8930 & 1.8414 & 1.7916 & 1.9250\\ 1.9087 & 1.8994 & 1.8668 & 1.9321\\ 1.8724 & 1.9298 & 1.9452 & 1.8302\\ 1.9484 & 1.9368 & 1.8426 & 1.8620 \end{array}]$$

$$T^{3}=[\begin{array}{cccc} 1.8824 & 1.7820 & 1.8736 & 1.9141\\ 1.9561 & 1.9490 & 1.8849 & 1.9409\\ 1.8703 & 1.9259 & 1.9216 & 1.8327\\ 1.9150 & 1.9548 & 1.7554 & 1.8069 \end{array}]$$

**Step 4:** The power weight of *D*_*k*_ associated with the IVq-ROULV $\alpha_{ij}^{k}$ with respect to the weight *γ*_*k*_ can be calculated by Eq (55). For convenience, $\delta_{ij}^{k}$ can be expressed by *δ*^*k*^, where *i*,*j* = 1,2,3,4;*k* = 1,2,3. Then, we can obtain

$$\delta^{1}=[\begin{array}{cccc} 0.2976 & 0.2966 & 0.3021 & 0.3019\\ 0.3015 & 0.3019 & 0.2945 & 0.3011\\ 0.2959 & 0.2968 & 0.3006 & 0.2909\\ 0.2971 & 0.3012 & 0.2942 & 0.3041 \end{array}]$$

$$\delta^{2}=[\begin{array}{cccc} 0.4020 & 0.4056 & 0.3939 & 0.3995\\ 0.3964 & 0.3960 & 0.4020 & 0.3989\\ 0.4024 & 0.4021 & 0.4010 & 0.4051\\ 0.4036 & 0.3983 & 0.4087 & 0.4010 \end{array}]$$

$$\delta^{3}=[\begin{array}{cccc} 0.3004 & 0.2978 & 0.3041 & 0.2985\\ 0.3021 & 0.3021 & 0.3034 & 0.3000\\ 0.3016 & 0.3011 & 0.2984 & 0.3041\\ 0.2993 & 0.3005 & 0.2971 & 0.2949 \end{array}]$$

**Step 5:** Utilize the IVq-ROULPWA operator to obtain the collective decision matrix of all DMs and the result is shown in Table 5.

**Step 6:** For Table 5, utilize the Eq (57) to obtain the *Sup*(*α*_*il*_,*α*_*if*_). For convenience, *Sup*(*α*_*il*_,*α*_*if*_) can be expressed by the symbol *S*^*lf*^, where *i*,*l*,*f* = 1,2,3,4;*l*≠*f*. Then, we can get

$$S^{12}=S^{21}=(\begin{array}{cccc} 0.9723 & 0.9302 & 0.9598 & 0.9670 \end{array}),\;S^{13}=S^{31}=(\begin{array}{cccc} 0.9705 & 0.9689 & 0.9410 & 0.9725 \end{array})$$

$$S^{14}=S^{41}=(\begin{array}{cccc} 0.9710 & 0.9705 & 0.9177 & 0.9597 \end{array}),\;S^{23}=S^{32}=(\begin{array}{cccc} 0.9516 & 0.9422 & 0.9869 & 0.9454 \end{array})$$

$$S^{24}=S^{42}=(\begin{array}{cccc} 0.9572 & 0.9550 & 0.9659 & 0.9402 \end{array}),\;S^{34}=S^{43}=(\begin{array}{cccc} 0.9391 & 0.9839 & 0.9787 & 0.9784 \end{array})$$

**Step 7:** Utilize Eq (58) to obtain the support *T*(*α*_*ij*_). Same as above, *T*(*α*_*ij*_) are expressed by *T*_*ij*_. Then, we can obtain

$$T=[\begin{array}{cccc} 2.9138 & 2.8697 & 2.8185 & 2.8993\\ 2.8812 & 2.8274 & 2.9126 & 2.8527\\ 2.8612 & 2.8950 & 2.9066 & 2.8964\\ 2.8673 & 2.9094 & 2.8622 & 2.8784 \end{array}]$$

**Step 8:** The power weight *η*_*ij*_ with respect to the IVq-ROULV *α*_*if*_ can be calculated by Eq (59). Then, we can obtain

$$\eta =[\begin{array}{cccc} 0.2723 & 0.2693 & 0.2665 & 0.2711\\ 0.1900 & 0.1874 & 0.1921 & 0.1885\\ 0.2089 & 0.2108 & 0.2120 & 0.2107\\ 0.3288 & 0.3325 & 0.3294 & 0.3296 \end{array}]$$

**Step 9:** For alternative *A*_*i*_(*i* = 1,2,3,4), calculate their comprehensive evaluation value *α*_*i*_(*i* = 1,2,3,4) by the IVq-ROULPWMM operator. Then, we can obtain (suppose that *H* = (1,1,1,1))

$$\alpha_{1}=\langle \left[s_{3.3684},s_{5.4831}],(\left[0.5299,0.6995],[0.9976,0.9997\right]\right)\rangle$$

$$\alpha_{2}=\langle \left[s_{3.3420},s_{5.6452}],(\left[0.4581,0.6490],[0.9986,0.9998\right]\right)\rangle$$

$$\alpha_{3}=\langle \left[s_{3.3288},s_{5.4401}],(\left[0.4563,0.6311],[0.9982,0.9999\right]\right)\rangle$$

$$\alpha_{4}=\langle \left[s_{3.2938},s_{4.8520}],(\left[0.5092,0.6414],[0.9990,0.9999\right]\right)\rangle$$

**Step 10:** Calculate the score values *S*(*α*_*i*_)(*i* = 1,2,3,4)

$$S(\alpha_{1})=0.0920\;S(\alpha_{2})=0.0701\;S(\alpha_{3})=0.0643\;S(\alpha_{4})=0.0678$$

**Step 11:** According to step 10, we can obtain the ranking order *A*_1_≻*A*_2_≻*A*_4_≻*A*_3_. Therefore, *A*_1_ is the best alternative.

**Table 5:** The collective interval-valued q-rung orthopair uncertain linguistic decision matrix of Example 5.

	***C***_**1**_	***C***_**2**_
*A*_1_	$\left\langle \left[s_{2.7292},s_{4.6090}],(\left[0.5064,0.7152],[0.1231,0.2259\right]\right)\right\rangle$	$\left\langle \left[s_{3.5435},s_{6.0000}],(\left[0.6000,0.7016],[0.1627,0.2659\right]\right)\right\rangle$
A_2_	$\left\langle \left[s_{3.0000},s_{4.7670}],(\left[0.4662,0.5658],[0.1906,0.2632\right]\right)\right\rangle$	$\left\langle \left[s_{3.8821},s_{6.0000}],(\left[0.4000,0.7465],[0.1233,0.2000\right]\right)\right\rangle$
A_3_	$\left\langle \left[s_{3.2207},s_{6.0000}],(\left[0.3851,0.5396],[0.1519,0.3104\right]\right)\right\rangle$	$\left\langle \left[s_{2.4369},s_{4.6145}],(\left[0.4359,0.6578],[0.1385,0.2457\right]\right)\right\rangle$
A_4_	$\left\langle \left[s_{3.8406},s_{6.0000}],(\left[0.5140,0.6350],[0.2000,0.3000\right]\right)\right\rangle$	$\left\langle \left[s_{2.5011},s_{3.7402}],(\left[0.4660,0.5656],[0.2656,0.3669\right]\right)\right\rangle$
	***C***_**3**_	***C***_**4**_
*A*_1_	$\left\langle \left[s_{4.0996},s_{6.0000}],(\left[0.4897,0.6419],[0.1541,0.2411\right]\right)\right\rangle$	$\left\langle \left[s_{3.4487},s_{4.7701}],(\left[0.5458,0.7757],[0.1000,0.2000\right]\right)\right\rangle$
A_2_	$\left\langle \left[s_{3.7382},s_{6.0000}],(\left[0.5145,0.6470],[0.1321,0.2653\right]\right)\right\rangle$	$\left\langle \left[s_{3.0000},s_{6.0000}],(\left[0.4749,0.6657],[0.1232,0.2260\right]\right)\right\rangle$
A_3_	$\left\langle \left[s_{4.0690},s_{6.0000}],(\left[0.4749,0.6749],[0.1230,0.2257\right]\right)\right\rangle$	$\left\langle \left[s_{4.4395},s_{6.0000}],(\left[0.5628,0.6818],[0.1324,0.2357\right]\right)\right\rangle$
A_4_	$\left\langle \left[s_{4.0789},s_{6.0000}],(\left[0.5016,0.6928],[0.1226,0.2253\right]\right)\right\rangle$	$\left\langle \left[s_{3.3381},s_{4.3698}],(\left[0.5808,0.7074],[0.1320,0.2353\right]\right)\right\rangle$

### 7.2 Sensitivity analysis

As mentioned in Section 4, parameters *H* and *q* occupy important positions in the final results. Besides, the final decision-making results also depend on the LSF. Next, we will discuss the effects of different parameters on the final result separately.

#### 7.2.1 The effect of the parameter q on the decision results

In the following, we attempt to reveal the influence of the parameter *q* on the decision results. To this end, we assign different values to *q* in the IVq-ROULPWMM operator and present the score values and decision results in Table 6. We assume *H* = (1,1,1,1) and LSF 1 is employed in the calculation process.

**Table 6:** Ranking results with different parameter values *q* when *H* = (1,1,1,1) based on LSF 1.

Parameters	Score functions *S*(*α*_*i*_)(*i* = 1,2,3,4)	Ranking orders
*q* = 2	*S*(*α*_1_) = 0.1877, *S*(*α*_2_) = 0.1635, *S*(*α*_3_) = 0.1538, *S*(*α*_4_) = 0.1437	*A*_1_≻*A*_2_≻*A*_3_≻*A*_4_
*q* = 3	*S*(*α*_1_) = 0.0920, *S*(*α*_2_) = 0.0701, *S*(*α*_3_) = 0.0643, *S*(*α*_4_) = 0.0678	*A*_1_≻*A*_2_≻*A*_4_≻*A*_3_
*q* = 4	*S*(*α*_1_) = 0.0608, *S*(*α*_2_) = 0.0441, *S*(*α*_3_) = 0.0389, *S*(*α*_4_) = 0.0417	*A*_1_≻*A*_2_≻*A*_4_≻*A*_3_
*q* = 5	*S*(*α*_1_) = 0.0419, *S*(*α*_2_) = 0.0278, *S*(*α*_3_) = 0.0248, *S*(*α*_4_) = 0.0264	*A*_1_≻*A*_2_≻*A*_4_≻*A*_3_
*q* = 6	*S*(*α*_1_) = 0.0294, *S*(*α*_2_) = 0.0171, *S*(*α*_3_) = 0.0162, *S*(*α*_4_) = 0.0170	*A*_1_≻*A*_2_≻*A*_4_≻*A*_3_
*q* = 7	*S*(*α*_1_) = 0.0209, *S*(*α*_2_) = 0.0116, *S*(*α*_3_) = 0.0109, *S*(*α*_4_) = 0.0111	*A*_1_≻*A*_2_≻*A*_4_≻*A*_3_
*q* = 8	*S*(*α*_1_) = 0.0151, *S*(*α*_2_) = 0.0077, *S*(*α*_3_) = 0.0075, *S*(*α*_4_) = 0.0074	*A*_1_≻*A*_2_≻*A*_3_≻*A*_4_
*q* = 9	*S*(*α*_1_) = 0.0110, *S*(*α*_2_) = 0.0051, *S*(*α*_3_) = 0.0052, *S*(*α*_4_) = 0.0049	*A*_1_≻*A*_3_≻*A*_2_≻*A*_4_

As we can see from Table 6, the optimal alternative by different values of parameter *q* is the same, i.e., *A*_1_, which means that hospital *A*_*1*_ is the most suitable one for patients to transfer to. In addition, we note that the increase of parameter *q* in the IVq-ROULPWMM operator leads to the decrease of the score values of comprehensive evaluation values. Therefore, we reckon that the parameter *q* has significant impacts on the decision results and how to choose a suitable value is an important problem. In [55], Liu and Wang illustrated that the smallest integer that guarantees *μ*^*q*^+*v*^*q*^≤1(*q*≥1) can be assigned to the value of parameter *q*, where *μ* represent the MD and *v* represent the NMD. In this paper, motivated by the method of selecting a proper value of *q* given by Liu and Wang [55], we provide a similar method for the appropriate assignment of the value of *q*. Let *α* = 〈[*s*_*θ*_,*s*_*τ*_],([*a*,*b*],[*c*,*d*])〉 be an IVq-ROULV provided by DMs for an attribute value, then the parameter *q* should be taken as the smallest integer that makes *b*^*q*^+*d*^*q*^≤1 hold. For instance, if a decision specialist provides *α* = 〈[*s*_2_,*s*_4_],([0.45,0.75],[0.55,0.95])〉 as the evaluation value, then the value of *q* can be taken 6, as 0.75^5^ + 0.95^5^ = 1.0111 > 1 and 0.75^6^ + 0.95^6^ = 0.9131 < 1.

#### 7.2.2 The influence of the parameter vector h on the decision results

In following, we attempt to study the influence of the parameter vector *H* on the decision results. Suppose that *q* = 3 and the calculation process is based on LSF 1. We assign different parameter vector in *H* and present the decision results in Table 7.

**Table 7:** Ranking results with different parameter vectors *H* when *q* = 3 based on LSF 1.

Parameters	Score functions *S*(*α*_*i*_)(*i* = 1,2,3,4)	Ranking orders
*H* = (1,0,0,0)	*S*(*α*_1_) = 0.1050, *S*(*α*_2_) = 0.0760, *S*(*α*_3_) = 0.0779, *S*(*α*_4_) = 0.0876	*A*_1_≻*A*_4_≻*A*_3_≻*A*_2_
*H* = (1,1,0,0)	*S*(*α*_1_) = 0.1001, *S*(*α*_2_) = 0.0743, *S*(*α*_3_) = 0.0722, *S*(*α*_4_) = 0.0825	*A*_1_≻*A*_4_≻*A*_2_≻*A*_3_
*H* = (1,1,1,0)	*S*(*α*_1_) = 0.0932, *S*(*α*_2_) = 0.0697, *S*(*α*_3_) = 0.0662, *S*(*α*_4_) = 0.0714	*A*_1_≻*A*_4_≻*A*_2_≻*A*_3_
*H* = (1,1,1,1)	*S*(*α*_1_) = 0.0920, *S*(*α*_2_) = 0.0701, *S*(*α*_3_) = 0.0643, *S*(*α*_4_) = 0.0678	*A*_1_≻*A*_2_≻*A*_4_≻*A*_3_

From Table 7, we can find that different score values and ranking orders are derived with different parameter vector *H* in the IVq-ROULPWMM operator. It is noted that the parameter vector *H* denotes the number of related attributes. To make it more convenient, we utilize *N*_*H*_(*N*_*H*_ = 1,2,3,4) to represent the number of connected attributes. When *N*_*H*_ = 1, our proposed method can only apply to decision-making scenarios where the attributes are independent of each other. When *N*_*H*_ = 2, 3 or 4, our proposed method is effective to deal with MAGDM problems where attributes are correlative. More concretely, when *N*_*H*_ = 2, then the proposed method will be able to handle application scenarios where there is a correlation between any two attributes. When *N*_*H*_ = 3, our method can cope with the interrelationships between any three attributes. When *N*_*H*_ = 4, the interrelationship among all the four is considered. In actual applications, DMs can select the most appropriate parameter vector *H* according to actual conditions.

#### 7.2.3 The influence of LSF on the results

It should be noted that, for convenience, we utilize the LFS1 to participate in the calculation process. To further explore the role of LSFs, we employ the other two LSFs, LSF2 and LSF3, to participate the calculation process, respectively. The score functions and ranking results calculating by three different LSF types are shown in Table 8. From Table 8, we can find that different score values and ranking results can be obtained with different types of LSF. In addition, the best alternative of LSF1 and LSF3 keeps the same, i.e., *A*_1_. However, the result of the second type of LSF is *A*_2_≻*A*_1_≻*A*_3_≻*A*_4_, which illustrates those types of LSF do have an influence on the decision results. In practical MAGDM problems, DMs can determine which LSF to utilize according to their personal preferences and actual application environment.

**Table 8:** Score functions and ranking orders by different LSFs when *q* = 3 and *H* = (1,1,1,1).

Parameters	Score functions *S*(*α*_*i*_)(*i* = 1,2,3,4)	Ranking orders
Our method based on LSF1 (*t* = 3)	*S*(*α*_1_) = 0.0920, *S*(*α*_2_) = 0.0701, *S*(*α*_3_) = 0.0643, *S*(*α*_4_) = 0.0678	*A*_1_≻*A*_2_≻*A*_4_≻*A*_3_
Our method based on LSF2 (*t* = 3, *ρ* = 1.37)	*S*(*α*_1_) = 0.5250, *S*(*α*_2_) = 0.5338, *S*(*α*_3_) = 0.5131, *S*(*α*_4_) = 0.4634	*A*_2_≻*A*_1_≻*A*_3_≻*A*_4_
Our method based on LSF3 (*t* = 3, *ε* = *β* = 0.5)	*S*(*α*_1_) = 0.5680, *S*(*α*_2_) = 0.5503, *S*(*α*_3_) = 0.5317, *S*(*α*_4_) = 0.5058	*A*_1_≻*A*_2_≻*A*_3_≻*A*_4_

### 7.3 Validity analysis

To verify the validity of the proposed method, we compare our method based on IVq-ROULPWMM operator with that proposed by Liu [28] based on IVIUL weighted geometric average (IVIULWGA) operator, and that proposed by Gao and Wei [29] based on IVPUL weighted average (IVPULWA) operator. The above methods are utilized to solve the example given in reference [28], which is briefly described as follows.

**Example 6.** (Adapted from Ref. [28]) After appraisal, the government decides to adopt five evaluation indexes: development of production *C*_1_, affluent living *C*_2_, rural civilization *C*_3_, clean and tidy village *C*_4_, and democratic management *C*_5_ to evaluate the new rural developing level of four secondary cites of Shandong province in China. Three experts are invited to investigate four secondary cities, they are, Weifan *A*_1_, Yantai *A*_2_, Binzhou *A*_3_ and Liaocheng *A*_4_. The weight of the experts is *ϖ* = (0.4,0.3,0.3)^*T*^, the weight of the attributes is *w* = (0.28,0.31,0.18,0.14,0.09)^*T*^, and the LTS *S* = (*s*_0_,*s*_1_,*s*_2_,*s*_3_,*s*_4_,*s*_5_,*s*_6_) are employed. The evaluation decisions of three DMs are listed in Tables 9–11, and the final results calculated by three different methods are shown in Table 12. From Table 12, we can find that although the score values calculated by different methods are slightly different from each other, the optimal alternative is always the same, i.e., *A*_1_, which demonstrate the validity and rationality of the proposed method.

**Table 9:** The interval-valued q-rung orthopair uncertain linguistic decision matrix *R*^1^ given by *D*_1_.

	*C*_1_	*C*_2_	C_3_	*C*_4_	*C*_5_
*A*_1_	<[*s*_4_, *s*_5_], ([0.7, 0.8], [0.1, 0.2])>	<[*s*_5_, *s*_5_], ([0.6, 0.6], [0.1, 0.3])>	<[*s*_5_, *s*_6_], ([0.8, 0.8], [0.1, 0.1])>	<[*s*_4_, *s*_4_], ([0.8, 0.8], [0.1, 0.1])>	<[*s*_5_, *s*_5_], ([0.7, 0.8], [0.1, 0.2])>
A_2_	<[*s*_5_, *s*_5_], ([0.6, 0.6], [0.1, 0.2])>	<[*s*_5_, *s*_6_], ([0.7, 0.7], [0.2, 0.2])>	<[*s*_4_, *s*_5_], ([0.5, 0.6], [0.2, 0.3])>	<[*s*_4_, *s*_5_], ([0.5, 0.6], [0.3, 0.3])>	<[*s*_4_, *s*_5_], ([0.9, 0.9], [0.0, 0.1])>
A_3_	<[*s*_4_, *s*_4_], ([0.7, 0.7], [0.2, 0.2])>	<[*s*_4_, *s*_4_], ([0.7, 0.8], [0.1, 0.2])>	<[*s*_5_, *s*_5_], ([0.7, 0.7], [0.1, 0.2])>	<[*s*_5_, *s*_5_], ([0.7, 0.8], [0.1, 0.2])>	<[*s*_3_, *s*_4_], ([0.8, 0.8], [0.1, 0.1])>
A_4_	<[*s*_3_, *s*_4_], ([0.6, 0.7], [0.2, 0.3])>	<[*s*_3_, *s*_3_], ([0.5, 0.6], [0.2, 0.3])>	<[*s*_4_, *s*_4_], ([0.6, 0.7], [0.2, 0.3])>	<[*s*_3_, *s*_4_], ([0.7, 0.7], [0.2, 0.2])>	<[*s*_5_, *s*_6_], ([0.6, 0.8], [0.3, 0.3])>

**Table 10:** The interval-valued q-rung orthopair uncertain linguistic decision matrix *R*^2^ given by *D*_2_.

	*C*_1_	*C*_2_	C_3_	*C*_4_	*C*_5_
*A*_1_	<[*s*_5_, *s*_6_], ([0.6, 0.7], [0.1, 0.1])>	<[*s*_5_, *s*_5_], ([0.7, 0.7], [0.1, 0.1])>	<[*s*_4_, *s*_5_], ([0.9, 0.9], [0.0, 0.1])>	<[*s*_5_, *s*_5_], ([0.7, 0.8], [0.1, 0.2])>	<[*s*_4_, *s*_6_], ([0.6, 0.6], [0.1, 0.1])>
A_2_	<[*s*_5_, *s*_5_], ([0.5, 0.7], [0.2, 0.2])>	<[*s*_4_, *s*_5_], ([0.6, 0.7], [0.2, 0.2])>	<[*s*_5_, *s*_4_], ([0.7, 0.7], [0.1, 0.2])>	<[*s*_6_, *s*_6_], ([0.6, 0.7], [0.1, 0.1])>	<[*s*_5_, *s*_5_], ([0.8, 0.9], [0.1, 0.1])>
A_3_	<[*s*_5_, *s*_5_], ([0.6, 0.7], [0.0, 0.2])>	<[*s*_4_, *s*_5_], ([0.8, 0.9], [0.1, 0.1])>	<[*s*_4_, *s*_4_], ([0.6, 0.6], [0.2, 0.2])>	<[*s*_4_, *s*_4_], ([0.7, 0.7], [0.2, 0.2])>	<[*s*_5_, *s*_5_], ([0.7, 0.8], [0.1, 0.2])>
A_4_	<[*s*_5_, *s*_5_], ([0.7, 0.8], [0.1, 0.2])>	<[*s*_4_, *s*_4_], ([0.5, 0.6], [0.2, 0.3])>	<[*s*_3_, *s*_3_], ([0.9, 0.9], [0.0, 0.1])>	<[*s*_3_, *s*_4_], ([0.8, 0.8], [0.1, 0.2])>	<[*s*_4_, *s*_4_], ([0.8, 0.8], [0.1, 0.1])>

**Table 11:** The interval-valued q-rung orthopair uncertain linguistic decision matrix *R*^3^ given by *D*_3_.

	*C*_1_	*C*_2_	C_3_	*C*_4_	*C*_5_
*A*_1_	<[*s*_5_, *s*_5_], ([0.7, 0.8], [0.1, 0.1])>	<[*s*_5_, *s*_5_], ([0.8, 0.9], [0.1, 0.1])>	<[*s*_5_, *s*_5_], ([0.8, 0.9], [0.1, 0.1])>	<[*s*_5_, *s*_6_], ([0.7, 0.8], [0.2, 0.2])>	<[*s*_4_, *s*_4_], ([0.8, 0.8], [0.1, 0.1])>
A_2_	<[*s*_5_, *s*_6_], ([0.6, 0.7], [0.1, 0.2])>	<[*s*_5_, *s*_6_], ([0.7, 0.7], [0.1, 0.2])>	<[*s*_5_, *s*_5_], ([0.8, 0.8], [0.1, 0.1])>	<[*s*_5_, *s*_5_], ([0.9, 0.9], [0.1, 0.1])>	<[*s*_5_, *s*_5_], ([0.8, 0.8], [0.2, 0.2])>
A_3_	<[*s*_5_, *s*_5_], ([0.8, 0.8], [0.0, 0.1])>	<[*s*_5_, *s*_5_], ([0.7, 0.8], [0.1, 0.2])>	<[*s*_4_, *s*_4_], ([0.7, 0.8], [0.1, 0.2])>	<[*s*_4_, *s*_4_], ([0.7, 0.8], [0.1, 0.1])>	<[*s*_4_, *s*_4_], ([0.8, 0.9], [0.0, 0.1])>
A_4_	<[*s*_4_, *s*_5_], ([0.8, 0.9], [0.1, 0.1])>	<[*s*_4_, *s*_4_], ([0.8, 0.8], [0.2, 0.2])>	<[*s*_4_, *s*_5_], ([0.8, 0.8], [0.0, 0.1])>	<[*s*_5_, *s*_5_], ([0.7, 0.7], [0.1, 0.2])>	<[*s*_4_, *s*_5_], ([0.7, 0.8], [0.1, 0.1])>

**Table 12:** Score functions and ranking orders by different methods of Example 6.

Methods	Score functions *S*(*α*_*i*_)(*i* = 1,2,3,4)	Ranking orders
Liu’s [28] method based on IVIULWGA operator	*S*(*α*_1_) = 3.9605, *S*(*α*_2_) = 3.7513 *S*(*α*_3_) = 3.5273, *S*(*α*_4_) = 2.9553	*A*_1_≻*A*_2_≻*A*_3_≻*A*_4_
Gao and Wei’s [29] method based on IVPULWA operator	*S*(*α*_1_) = 3.8915, *S*(*α*_2_) = 3.7304 *S*(*α*_3_) = 3.4509, *S*(*α*_4_) = 3.0340	*A*_1_≻*A*_2_≻*A*_3_≻*A*_4_
The proposed method based on IVq-ROULPWMM operator (*q* = 1, *H* = (1,0,0,0,0))	*S*(*α*_1_) = 0.1764, *S*(*α*_2_) = 0.1336 *S*(*α*_3_) = 0.1408, *S*(*α*_4_) = 0.1291	*A*_1_≻*A*_3_≻*A*_2_≻*A*_4_

### 7.4 Advantages of our proposed method

To illustrate the superiorities of the proposed method, we compare it with Liu’s [28] method based on IVIULWGA operator, and Gao and Wei’s [29] method based on IVPULWA operator. We utilize the above-mentioned methods to deal with the following numerical examples and compare their ranking results to explain the advantages and superiorities of the proposed method.

#### 7.4.1 The larger information space for DMs to express their preference information

As aforementioned, our proposed method is based on IVq-ROULVs. As analyzed above, IVIULSs and IVPULSs can be regard as two special cases of IVq-ROULs. When *q* = 1, IVq-ROULSs reduce to the IVIULSs. When *q* = 2, IVq-ROULSs reduce to IVPULSs. Compared with IVIULSs and IVPULSs, IVq-ROULSs have more permissive rules and can provide larger information space for DMs in the process of providing their evaluation information. To describe this advantage more clearly, we give the following example.

**Example 7.** In Example 5, suppose that for some reasons, DM *D*_2_ prefers to utilize *α*′ = 〈[*s*_2_,*s*_3_],([0.7,0.9],[0.4,0.5])〉 as the evaluation value on attribute *C*_2_ of *A*_1_. The other evaluation values remain unchanged. We use above mentioned decision-making methods to solve Example 7 and present the decision results on Table 13.

**Table 13:** Score functions and ranking orders by different methods of Example 7.

Methods	Score functions *S*(*α*_*i*_)(*i* = 1,2,3,4)	Ranking orders
Liu’s [28] method based on IVIULWGA operator	Cannot be calculated	—
Gao and Wei’s [29] method based on IVPULWA operator	Cannot be calculated	—
The proposed method based on IVq-ROULPWMM operator (*q* = 3, *H* = (1,1,1,1))	*S*(*α*_1_) = 0.0898, *S*(*α*_2_) = 0.0701 *S*(*α*_3_) = 0.0643, *S*(*α*_4_) = 0.0678	*A*_1_≻*A*_2_≻*A*_4_≻*A*_3_

It is noted that 0.9+0.5 = 1.4>1and 0.9^2^+0.5^2^ = 1.06>1, Either IVIULSs or IVPULSs is inappropriate to express *α*′. In this case, IVq-ROULSs cannot be more suitable to represent, as 0.9^3^ + 0.5^3^ = 0.845 < 1 (i.e., *q* = 3). As a result, the methods proposed in [28,29] are ineffective to handle Example 7, while our method can still effectively determine the optimal alternative. This example illustrates the advantage of our method, i.e., it provides larger information space for DMs to fully express their evaluation information.

#### 7.4.2 The ability of reducing the negative impact of extreme evaluation values on the final decision results

In most MAGDM problems, DMs usually have different experience and personalities. In addition, due the high complexity and time shortage, the information available to experts is not comprehensive enough. Hence, DMs are likely to provide some extremely or unreasonable evaluation values, which may have negative impacts on the decision. As mentioned in Section 5, our proposed MAGDM is based on the PA and PMM operator. The PA operator allows argument values to support each other in the aggregation process. Hence, the PA operator can effectively deal with DMs’ extreme evaluation values, making the final results more reasonable. To better illustrate this advantage, we provide the following example.

**Example 8.** Assume that the DM *D*_2_ in Example 6 prefers alternative *A*_2_ and DM *D*_2_ is biased against *A*_1_. For the evaluation value of attribute *C*_3_ of *A*_2_, DM *D*_2_ provides a high value 〈[*s*_5_,*s*_6_],([0.8,0.9],[0.1,0.1])〉 for his/her evaluation information. For attribute *C*_2_ of *A*_1_, DM *D*_1_ employs an IVq-ROULV 〈[*s*_1_,*s*_2_],([0.1,0.2],[0.8,0.9])〉 as his/her evaluation values. The other attribute values remain unchanged. We use our decision-making method and those presented in [28,29] to solve Example 8, and results are listed in Table 14.

**Table 14:** Score functions and ranking orders by different methods of Example 8.

Methods	Score functions *S*(*α*_*i*_)(*i* = 1,2,3,4)	Ranking orders
Liu’s [28] method based on IVIULWGA operator	*S*(*α*_1_) = 2.7911, *S*(*α*_2_) = 3.8193 *S*(*α*_3_) = 3.5273, *S*(*α*_4_) = 2.9553	*A*_2_≻*A*_3_≻*A*_4_≻*A*_1_
Gao and Wei’s [29] method based on IVPULWA operator	*S*(*α*_1_) = 3.4928, *S*(*α*_2_) = 3.8173 *S*(*α*_3_) = 3.4509, *S*(*α*_4_) = 3.0340	*A*_2_≻*A*_1_≻*A*_3_≻*A*_4_
The proposed method based on IVq-ROULPWMM operator (*q* = 1, *H* = (1,0,0,0,0))	*S*(*α*_1_) = 0.1716, *S*(*α*_2_) = 0.1417 *S*(*α*_3_) = 0.1408, *S*(*α*_4_) = 0.1291	*A*_1_≻*A*_2_≻*A*_3_≻*A*_4_

From Table 14, it is noted that the ranking order produced by Liu’s [28] method changes from *A*_1_≻*A*_2_≻*A*_3_≻*A*_4_ to *A*_2_≻*A*_3_≻*A*_4_≻*A*_1_, which indicates *A*_2_ rather *A*_1_ is the optimal alternative. The ranking result derived by Gao and Wei’s [29] method changes from *A*_1_≻*A*_2_≻*A*_3_≻*A*_4_ to *A*_2_≻*A*_1_≻*A*_3_≻*A*_4_, and *A*_2_ is the optimal alternative. However, the optimal alternative produced by our proposed method is still *A*_1_. In Example 8, DM *D*_2_ provides some unduly high and DM *D*_1_ provides some unduly low evaluation values. Due to the biased evaluation values, the ranking orders derived by Liu [28] and Gao and Wei’s [29] have changed, and the optimal alternative changes from *A*_1_ to *A*_2_. This is because Liu’s [28] and Gao and Wei’s [29] methods fail to reasonably figure out DMs’ unreasonable evaluations. In addition, our method is based PA and PMM operators, so that it can reduce the bad influences of unreasonable evaluation values on the decision results. In other words, the decision results produced by the proposed method are more robust and dependable.

#### 7.4.3 The ability of capturing the interrelationship among any numbers of attributes

In most real MAGDM methods, the attributes are not independent with each other. For instance, in Example 5, the attributes *C*_1_ (doctor’s level of medical satisfaction), *C*_2_ (medical facility satisfaction) and *C*_3_ (hospital drug supply satisfaction) have interactions. Generally, better medical facility means better ability of hospital drug supply, and higher medical level. Therefore, when calculating the overall evaluation values of alternatives, it is necessary to take the interrelationship among attributes into consideration. However, Liu’s [28] and Gao and Wei’s [29] methods are based on the simple weighted average or weighted geometric average operators and have no power to consider the interactive relationship among attributes, which maybe result in unreasonable decision results. Our method is based on the PMM operator, which has the capability of reflecting the interrelationship among attributes. In addition, our method is more flexible with the parameter vector *H*. If there is no relationship among any attributes, then we can set (1,0,0,0) to the parameter vector *H*. If *H* = (1,1,0,0), then we can solve any MAGDM situations where there is a correlation between any two attributes. If *H* = (1,1,1,0), our method can solve scenarios where such three attributes are related. If *H* = (1,1,1,1), then we can consider all the relationships between the input arguments. To sum up, our method is more powerful and flexible than those presented in [28,29].

#### 7.4.4 The flexibility of the calculation process

It is noted that operational results of IVIULVs proposed by Liu [28] directly based on the subscripts of LTS. The main drawback of these operations is that the calculation results are not closed and exceed the upper limit of the given LST. Additionally, the operations of IVPULVs proposed by Gao and Wei [29] have the similar shortcoming. We provide the following example to better demonstrate the shortcoming.

**Example 9.** Let *α*_1_ = 〈[*s*_2_,*s*_3_],([0.5,0.6],[0.2,0.3])〉 and *α*_2_ = 〈[*s*_4_,*s*_5_],([0.3,0.4],[0.4,0.5])〉 be two IVIULVs, then according to the operations proposed by Liu [44], we have

$$\alpha_{1}⊕\alpha_{2}=\langle \left[s_{6},s_{8}],(\left[0.65,0.76],[0.08,0.15\right]\right)\rangle ,\alpha_{1}⊗\alpha_{2}=\langle \left[s_{8},s_{15}],(\left[0.15,0.24],[0.52,0.65\right]\right)\rangle ,$$

$$3\alpha_{1}=\langle \left[s_{6},s_{9}],(\left[0.875,0.936],[0.008,0.027\right]\right)\rangle ,\alpha_{1}^{3}=\langle \left[s_{8},s_{27}],(\left[0.125,0.216],[0.488,0.657\right]\right)\rangle .$$

If we employ the operational rules proposed by Gao and Wei [29], then we have (It is noted that all IVIULVs are IVPULVs, and so that *α*_1_ and *α*_2_ are also IVPULVs. Hence, the operations proposed by Gao and Wei [29] are suitable for *α*_1_ and *α*_2_.)

$$\alpha_{1}⊕\alpha_{2}=\langle \left[s_{6},s_{8}],(\left[0.5635,0.68],[0.08,0.15\right]\right)\rangle ,\alpha_{1}⊗\alpha_{2}=\langle \left[s_{8},s_{15}],(\left[0.15,0.24],[0.44,0.5635\right]\right)\rangle ,$$

$$3\alpha_{1}=\langle \left[s_{6},s_{9}],(\left[0.7603,0.859],[0.008,0.027\right]\right)\rangle ,\alpha_{1}^{3}=\langle \left[s_{8},s_{27}],(\left[0.125,0.216],[0.3395,0.6845\right]\right)\rangle .$$

The operational rules of IVq-ROULVs proposed in the present paper are based on LSF. Hence, the operations derived by our proposed operational rules of IVq-ROULVs are still closed. In this Example, if we employ the operations of IVq-ROULVs, we can obtain (*q* = 1 and LSF1 is used)

$$\alpha_{1}⊕\alpha_{2}=\langle \left[4.6667,5.5],(\left[0.65,0.76],[0.08,0.15\right]\right)\rangle ,$$

$$\alpha_{1}⊗\alpha_{2}=\langle \left[1.3333,2.5],(\left[0.15,0.24],[0.52,0.65\right]\right)\rangle ,$$

$$3\alpha_{1}=\langle \left[4.2222,5.25],(\left[0.875,0.936],[0.008,0.027\right]\right)\rangle ,$$

$$\alpha_{1}^{3}=\langle \left[0.2222,0.75],(\left[0.125,0.216],[0.488,0.657\right]\right)\rangle .$$

The example reveals two prominent advantages of our proposed calculation process. First, the calculation process makes the results closed. Second, the operations guarantee that the evaluation information still satisfies the semantic conversion requirements after being assembled. Therefore, our proposed method can match different semantic translation requirements and DMs can determine which LSF to employ according to their personal preferences and actual application environment. In light of these reasons, our method is more powerful and flexible than Liu’s [28] and Gao and Wei’s [29] decision-making methods.

#### 7.4.5 The ability of depicting DMs’ evaluation values comprehensively

The IVq-ROULS is based on the combination of IVq-ROFS and ULV. Hence, our method can fully express DMs’ evaluation information. First, our method is better than those proposed by Joshi et al. [18] and Gao et al. [66]. This is because Joshi et al.’s [18] and Gao et al.’s [66] methods are based on IVq-ROFSs, which only describe DMs’ quantitative evaluation information. Our method is based on IVq-ROULSs, so it can describe both DMs’ quantitative and qualitative evaluation values. Hence, our method is more powerful than Joshi et al.’s [18] method and Gao et al.’s [66] method. Moreover, our method is also more powerful than those proposed in [24–27]. This is because the methods given in [24–27] are based on q-ROULSs and our method is based on IVq-ROULSs. Actually, the q-ROULS is a special case of IVq-ROULS and the q-rung orthopair uncertain linguistic variable (q-ROULV) is a special case of IVq-ROULV, where the upper and lower bound of MD is equal and the upper and lower bound of NMD is equal. For instance, let 〈[*s*_1_,*s*_2_],(0.5,0.9)〉 be a q-ROULV, then we can transform it into 〈[*s*_1_,*s*_2_],([0.5,0.5],[0.9,0.9])〉, which is an IVq-ROULV. In other words, our method can effectively deal with those MAGDM problems, which utilize q-ROULVs to represent the evaluation values. But the decision-making methods proposed in [25–27] are powerless to handle MAGDM problems with IVq-ROULVs. Hence, our method can fully describe DMs’ evaluation values.

### 7.5 Discussion on the obtained results

To give a further and comprehensive description on the advantages and superiors of the proposed method, we summarize the characteristics of some MAGDM methods in Table 15. Table 15 contains the common decision scenarios in MAGDM problems, and we can clearly find that there are always one or several situations that other operators cannot up to. By way of contrast, our proposed method is capable of handling all of this, it can not only provide DMs the greatest degree of decision-making freedom, but also consider the relationship between arbitrary attributes, it can also consider both qualitative and quantitative information simultaneously. All the above advantages make it have tremendous superiority in MAGDM problems.

**Table 15:** Characteristics of different MAGDM methods.

Method	Whether permits the square sum of MD and DM to be greater than one	Whether permits the sum of *q*th power of MD and *q*th power of DM to be greater than one	Whether eliminates the bad influence of unreasonable attribute values on the decision results	Whether considers the interrelationship among any two attributes	Whether takes the interrelationship among multiple attributes into account	Whether takes DMs’ qualitative information into account	Whether takes the interval-values MDs and NMDs into account
Wang et al.’s [24] method	Yes	Yes	No	Yes	Yes	Yes	No
Xing et al.’s [25] method	Yes	Yes	No	No	No	Yes	No
Liu et al.’s [26] method	Yes	Yes	No	No	No	Yes	No
Liu’s [28] method	No	No	No	No	No	Yes	Yes
Bai et al.’s [27] method	Yes	Yes	Yes	Yes	Yes	Yes	No
Gao and Wei’s [29] method	Yes	No	No	No	No	Yes	Yes
Gao et al.’s [66] method	Yes	Yes	No	Yes	Yes	No	Yes
The proposed method	Yes	Yes	Yes	Yes	Yes	Yes	Yes

## 8 Conclusions remarks

This paper introduced a new MAGDM method. We first proposed the concept of IVq-ROULSs by combining IVq-ROFSs with ULVs. Afterward, some other related notions, such as operational rules, comparison method, and distance measure were proposed based on LSF. Based on this, we proposed a family of IVq-ROUL aggregation operators to aggregate IVq-ROULVs. With the help of the proposed operators, we gave the main steps of solving MAGDM problems with IVq-ROUL information. We further showed the performance of the proposed method in the process of downward referral hospital evaluation and analyzed the impacts of the parameters on the decision results, the validity analysis illustrate the effectiveness of our method. To illustrate that why DMs should use our proposed method rather than other methods, we conducted several comparative analyses. Results show that the IVq-ROULS is an efficient tool to describe DMs’ evaluation values in MAGDM process quantificationally and qualitatively, and the proposed method can not only consider the interrelationships among multiple input arguments, but also eliminate the influences of unreasonable data on the final results. Besides, our proposed method has a powerful ability to alleviate the negative effects of unduly high or low evaluations.

Given the good performance of the proposed method, in future works, we shall apply it to more classical MAGDM problem, such as investment selection, low carbon selection, medical diagnosis, landfill site selection, airline service evaluation, etc. These problems require DMs to evaluate and make decisions on the multiple attributes with respect to the alternatives, which will inevitably produce qualitative and uncertain information. It has been verified that the method proposed in this paper has excellent advantages in dealing with similar problems, thus we can try to apply it to these problems in future works.

Although our proposed method has many advantages when comparing with the existing methods, it still constrained when faced with hesitant or dual hesitant evaluation information. In addition, the method proposed in this paper is based on DMs’ subjective evaluation, although it can alleviate the untruthfulness of the results caused by the extreme information to a certain extent, it is still powerless to confirm or correct the inaccuracy or inconsistent evaluation information. In future works, we should pay more attention to these limitations, and strengthen the depth of the research.

## References

[1] Liu S, Yu W, Liu L, Hu Y. Variable weights theory and its application to multi-attribute group decision-making with intuitionistic fuzzy numbers on determining decision maker’s weights. PLOS One. 2019;14(3): e0212636. doi: 10.1371/journal.pone.0212636 30840647 PMC6402660

[2] Liu P, Li D. Some Muirhead mean operators for intuitionistic fuzzy numbers and their applications to group decision-making. PLOS One. 2017;12(1): e0168767. doi: 10.1371/journal.pone.0168767 28103244 PMC5245779

[3] Sinani F, Erceg Z, Vasiljević M. An evaluation of a third-party logistics provider: The application of the rough Dombi-Hamy mean operator. Decision Making: Appl. Manage. Eng. 2020;3(1): 92–107.

[4] Ali Z, Mahmood T, Ullah K, Khan Q. Einstein geometric aggregation operators using a novel complex interval-valued Pythagorean fuzzy setting with application in green supplier chain management. Rep. Mech. Eng. 2021;2(1): 105–134.

[5] Ramakrishnan K, Chakraborty S. A cloud TOPSIS model for green supplier selection. Facta Universitatis, Series: Mech. Eng. 2020;18(3): 375–397.

[6] Xing Y, Zhang R, Wang J, Zhu X. Some new Pythagorean fuzzy Choquet-Frank aggregation operators for multi-attribute decision making. Int J Intell Syst. 2018;33(11): 2189–2215.

[7] Wang J, Shang X, Bai K, Xu Y. A new approach to cubic q-rung orthopair fuzzy multiple attribute group decision-making based on power Muirhead mean. Neural Comput Appl, online, 2020; doi: 10.1007/s00521-020-04807-9

[8] Riaz M, Çagman N, Wali N, Mushtaq A. Certain properties of soft multi-set topology with applications in multi-criteria decision making. Decision Making: Appl. Manage. Eng. 2020;3(2): 70–96.

[9] Pamucar D, Ecer F. Prioritizing the weights of the evaluation criteria under fuzziness: The fuzzy full consistency method–FUCOM-F. Facta Universitatis, Series: Mech. Eng. 2020;18(3): 419–437.

[10] Pamučar D, Janković A. The application of the hybrid interval rough weighted Power-Heronian operator in multi-criteria decision making. Operational Research in Engineering Sciences: Theor. Appl. 2020;3(2): 54–73.

[11] Zadeh LA. Fuzzy set theory. Inf Control. 1965;8(3): 338–353.

[12] Atanassov KT. Intuitionistic fuzzy sets. Fuzzy Sets Syst. 1986;20(1): 87–96.

[13] Yager RR. Pythagorean membership grades in multicriteria decision making. IEEE Trans Fuzzy Syst. 2014;22(4): 958–965.

[14] Yager RR. Generalized orthopair fuzzy sets. IEEE Trans Fuzzy Syst. 2017;25(5): 1222–1230.

[15] Zeng S, Hu Y, Xie X. Q-rung orthopair fuzzy weighted induced logarithmic distance measures and their application in multiple attribute decision making. Eng. Appl. Artif. Intel. 2021;100: 104167.

[16] Ali Z, Mahmood T. Maclaurin symmetric mean operators and their applications in the environment of complex q-rung orthopair fuzzy sets. Comput. Appl. Math. 2020;39: 1–27.

[17] Xu Y, Shang X, Wang J, Wu W, Huang H. Some q-rung dual hesitant fuzzy Heronian mean operators with their application to multiple attribute group decision-making. Symmetry. 2018;10(10): 472.

[18] Joshi BP, Singh A, Bhatt PK, Vaisla KS. Interval-valued q-rung orthopair fuzzy sets and their properties. J Intell Fuzzy Syst. 2018;35(5): 5225–5230.

[19] Xu Y, Shang X, Wang J, Zhao H, Zhang R, Bai K. Some interval-valued q-rung dual hesitant fuzzy Muirhead mean operators with their application to multi-attribute decision-making. IEEE Access. 2019;7: 54724–54745.

[20] Liu P, Liu W. Multiple-attribute group decision-making based on power Bonferroni operators of linguistic q-rung orthopair fuzzy numbers. Int J Intell Syst. 2019;34(4): 652–689.

[21] Li L, Zhang R, Shang X. Some q-rung orthopair linguistic Heronian mean operators with their application to multi-attribute group decision making. Arch Control Sci. 2018;28(4): 551–583.

[22] Wang H, Ju Y, Liu P. Multi-attribute group decision-making methods based on q-rung orthopair fuzzy linguistic sets. Int J Intell Syst. 2019;34(6): 1129–1157.

[23] Li L, Zhang R, Wang J, Shang X, Bai K. A novel approach to multi-attribute group decision-making with q-rung picture linguistic information. Symmetry. 2018;10(5): 172.

[24] Wang J, Zhang R, Li L, Zhu X, Shang X. A novel approach to multi-attribute group decision making based on q-rung orthopair uncertain linguistic information. J Intell Fuzzy Syst. 2019;36(6): 5565–5581.

[25] Xing Y, Zhang R, Zhu X, Bai K. q-Rung orthopair fuzzy uncertain linguistic choquet integral operators and their application to multi-attribute decision making. J Intell Fuzzy Syst. 2019;37(1): 1123–1139.

[26] Liu Z, Xu H, Yu Y, Li J. Some q-rung orthopair uncertain linguistic aggregation operators and their application to multiple attribute group decision making. Int J Intell Syst. 2019;34(10): 2521–2555.

[27] Bai K, Zhu Z, Wang J, Zhang R. Power partitioned Heronian mean operators for q-rung orthopair uncertain linguistic sets with their application to multi-attribute group decision making. Int J Intell Syst. 2020;35(1): 3–37.

[28] Liu P. Some geometric aggregation operators based on interval intuitionistic uncertain linguistic variables and their application to group decision making. Appl Math Model. 2013;37(4): 2430–2444.

[29] Gao H, Wei G. Multiple attribute decision making based on interval-valued Pythagorean uncertain linguistic aggregation operators. Int J Knowl-based Intell Eng Syst. 2018;22(1): 59–81.

[30] Yager RR. The power average operator. IEEE T Syst Man Cy A. 2001;31(6): 724–731.

[31] Liang D, Darko AP, Zeng J. Interval-valued Pythagorean fuzzy power average-based MULTIMOORA method for multi-criteria decision-making. J Exp Theor Artif In. 2019;1–30.

[32] Liu P, Li Y, Teng F. Bidirectional projection method for probabilistic linguistic multi-criteria group decision-making based on power average operator. Int J Fuzzy Syst. 2019;21(8): 2340–2353.

[33] Song Y, Deng Y. A new soft likelihood function based on power ordered weighted average operator. Int J Intell Syst. 2019;34(11): 2988–2999.

[34] Xiong SH, Chen ZS, Chang JP, Chin KS. On extended power average operators for decision-making: A case study in emergency response plan selection of civil aviation. Comput Ind Eng. 2019;130: 258–271.

[35] Li L, Zhang R, Wang J, Zhu X, Xing Y. Pythagorean fuzzy power Muirhead mean operators with their application to multi-attribute decision making. J Intell Fuzzy Syst. 2018;35(2): 2035–2050.

[36] Muirhead RF. Some methods applicable to identities and inequalities of symmetric algebraic functions of n letters. P Edinburgh Math Soc. 1903;21: 144–162.

[37] Liu Z, Li L, Wang X, Liu P. A novel multiple-attribute decision making method based on power Muirhead mean operator under normal wiggly hesitant fuzzy environment. J Intell Fuzzy Syst. 2019;37(5): 7003–7023.

[38] Liu P, Khan Q, Mahmood T, Hassan N. T-spherical fuzzy power Muirhead mean operator based on novel operational laws and their application in multi-attribute group decision making. IEEE Access. 2019;7: 22613–22632.

[39] Luo S, Liang W, Zhao G. Linguistic neutrosophic power Muirhead mean operators for safety evaluation of mines. PloS One. 2019;14(10): e0224090. doi: 10.1371/journal.pone.0224090 31648224 PMC6813029

[40] Xu W, Shang X, Wang J, Li W. A novel approach to multi-attribute group decision-making based on interval-valued intuitionistic fuzzy power Muirhead mean. Symmetry. 2019;11(3): 441.

[41] Liu P, Khan Q, Mahmood T. Some single-valued neutrosophic power Muirhead mean operators and their application to group decision making. J Intell Fuzzy Syst. 2019;37(2): 2515–2537.

[42] Liu P, Wang P. Some q-rung orthopair fuzzy aggregation operators and their applications to multiple-attribute decision making. Int J Intell Syst. 2018;33(2): 259–280.

[43] Xing Y, Zhang R, Zhou Z, Wang J. Some q-rung orthopair fuzzy point weighted aggregation operators for multi-attribute decision making. Soft Comput. 2019;23: 1–23.

[44] Yang W, Pang Y. New q-rung orthopair fuzzy partitioned Bonferroni mean operators and their application in multiple attribute decision making. Int J Intell Syst. 2018;34(3): 439–476.

[45] Liu P, Liu J. Some q-rung orthopair fuzzy Bonferroni mean operators and their application to multi-attribute group decision making. Int J Intell Syst. 2017;33(2): 315–347.

[46] Wei G, Gao H, Wei Y. Some q-rung orthopair fuzzy Heronian mean operators in multiple attribute decision making. Int J Intell Syst. 2018;33(7): 1426–1458.

[47] Wang J, Wei G, Lu J, Alsaadi FE, Hayat T, Wei C, et al. Some q-rung orthopair fuzzy Hamy mean operators in multiple attribute decision-making and their application to enterprise resource planning systems selection. Int J Intell Syst. 2019;34(10): 2429–2458.

[48] Liu Z, Wang S, Liu P. Multiple attribute group decision making based on q-rung orthopair fuzzy Heronian mean operators. Int J Intell Syst. 2018;33(12): 2341–2363.

[49] Wei G, Wei C, Wang J, Gao H, Wei Y. Some q-rung orthopair fuzzy Maclaurin symmetric mean operators and their applications to potential evaluation of emerging technology commercialization. Int J Intell Syst. 2019;34(1): 50–81.

[50] Bai K, Zhu X, Wang J, Zhang R. Some partitioned Maclaurin symmetric mean based on q-rung orthopair fuzzy information for dealing with multi-attribute group decision making. Symmetry 2018;10(9): 383.

[51] Wang J, Zhang R, Zhu X, Zhou Z, Shang X, Li W. Some q-rung orthopair fuzzy Muirhead means with their application to multi-attribute group decision making. J Intell Fuzzy Syst. 2019;36(2): 1599–1614.

[52] Liang D, Zhang Y, Cao W. q-Rung orthopair fuzzy Choquet integral aggregation and its application in heterogeneous multicriteria two-sided matching decision making. Int J Intell Syst. 2019;34(12): 3275–3301.

[53] Ju Y, Luo C, Ma J, Wang A. A novel multiple-attribute group decision-making method based on q-rung orthopair fuzzy generalized power weighted aggregation operators. Int J Intell Syst. 2019;34(9): 2077–2103.

[54] Jana C, Muhiuddin G, Pal M. Some Dombi aggregation of q-rung orthopair fuzzy numbers in multiple-attribute decision making. Int J Intell Syst. 2019;34(12): 3220–3240.

[55] Liu P, Wang P. Multiple-attribute decision-making based on Archimedean Bonferroni operators of q-rung orthopair fuzzy numbers. IEEE T Fuzzy Syst. 2018;27(5): 834–848.

[56] Xing Y, Zhang R, Wang J, Bai K, Xue J. A new multi-criteria group decision-making approach based on q-rung orthopair fuzzy interaction Hamy mean operators. Neural Comput Appl. 2019;4: 1–24.

[57] Peng X, Dai J, Garg H. Exponential operation and aggregation operator for q-rung orthopair fuzzy set and their decision-making method with a new score function. Int J Intell Syst. 2018;33(11): 2255–2282.

[58] Garg H. CN‐q‐ROFS: Connection number‐based q‐rung orthopair fuzzy set and their application to decision‐making process. Int J Intell Syst. 2021;36(7): 3106–3143.

[59] Garg H, Ali Z, Mahmood T. Algorithms for complex interval‐valued q‐rung orthopair fuzzy sets in decision making based on aggregation operators, AHP, and TOPSIS. Expert Syst. 2021;38(1): e12609.

[60] Zhang B, Mahmood T, Ahmmad J, Khan Q, Ali Z, Zeng S. Cubic q-Rung orthopair fuzzy Heronian mean operators and their applications to multi-attribute group decision making. Mathematics, 2020;8(7): 1125.

[61] Rong Y, Liu Y, Pei Z. Complex q‐rung orthopair fuzzy 2‐tuple linguistic Maclaurin symmetric mean operators and its application to emergency program selection. Int J Intell Syst. 2020;35(11): 1749–1790.

[62] Zhang H. (2014). Linguistic intuitionistic fuzzy sets and application in MAGDM. J Appl Math. 2014;Article ID: 432092.

[63] Garg H. Linguistic Pythagorean fuzzy sets and its applications in multi-attribute decision‐making process. Int J Intell Syst. 2018;33(6): 1234–1263.

[64] Zhang C, Tian G, Fathollahi-Fard AM, Wang W, Wu P, Li Z. Interval-valued intuitionistic uncertain linguistic cloud petri net and its application to risk assessment for subway fire accident. IEEE T Autom Sci Eng. 2020; doi: 10.1109/TASE.2020.3014907

[65] Liu HC, Quan MY, Shi H, Guo C. An integrated MCDM method for robot selection under interval‐valued Pythagorean uncertain linguistic environment. Int J Intell Syst. 2019;34(2): 188–214.

[66] Gao H, Ju Y, Zhang W, Ju D. Multi-attribute decision-making method based on interval-valued q-rung orthopair fuzzy Archimedean Muirhead mean operators. IEEE Access. 2019;7: 74300–74315.

[67] Wang J, Wu J, Wang J, Zhang H, Chen X. Interval-valued hesitant fuzzy linguistic sets and their applications in multi-criteria decision-making problems. Inform Sci. 2014;288: 55–72.

