# Expression of Concern: Multiple attribute group decision-making based on interval-valued q-rung orthopair uncertain linguistic power Muirhead mean operators and linguistic scale functions

**DOI:** 10.1371/journal.pone.0362713

**Published:** 2026-11-13

**Authors:** The PLOS One Editors

After this article [1] was published, the following concerns were noted:

- The articles cited as References 24, 29, 41, and 51 were retracted after [1] was published.
- Concerns have been raised on PubPeer about methodology reported in References 2, 4, 17, 19, 21-23, 31, 34-35, 39, 40, 43-44, 46-50, 56-57, 59, 64, and 67.

In light of the cumulative issues, the *PLOS One* Editors issue this Expression of Concern. Readers are advised to interpret the article [1] with caution.
